# Differential Reductions in Total and Compositional PM_2.5_ Exposure across Socioeconomic and Demographic Groups from Emission Source Mitigation in Canada (2007–2016)

**DOI:** 10.1021/acs.est.5c17598

**Published:** 2026-04-14

**Authors:** Anna Delic, Katarina Kunarac, Li Chen, Amanda Pappin, Aaron van Donkelaar, Randall Martin, Hong Chen

**Affiliations:** † Environmental Health Science & Research Bureau, 6348 Health Canada, Ottawa, Ontario K1A 0K9, Canada; ‡ Water and Air Quality Bureau, Health Canada, Ottawa, Ontario K1A 0K9, Canada; § Department of Energy, Environment & Chemical Engineering, 7548 Washington University, St. Louis, Missouri 63112, United States; ∥ Public Health Ontario, Toronto, Ontario M5G 1V2, Canada; ⊥ ICES, Toronto, Ontario M4N 3M5, Canada; # Dalla Lana School of Public Health, University of Toronto, Toronto, Ontario M5T 3M7, Canada

**Keywords:** PM_2.5_ total mass and components, socioeconomics and demographics, inequities, emissions reductions, Canada

## Abstract

Fine particulate matter (PM_2.5_) is a leading risk factor for morbidity and mortality worldwide. Recent studies have shown substantial heterogeneity in exposure to PM_2.5_ within populations, with socially disadvantaged subgroups exhibiting disproportionally higher exposure. However, the extent to which reductions in PM_2.5_ and its components may differentially affect these subgroups remains unclear. Leveraging state-of-the-art exposure surfaces that simulate annual reductions in total and compositional PM_2.5_ from major emission sectors under two policy scenarios (20% and 100%), based on satellite observations and a global chemical transport model, we characterized the expected reductions in total and compositional population-weighted PM_2.5_ exposure by selected subgroups for 2007 and 2016. Under both scenarios, we found that certain subgroups, particularly low-income people, immigrants, and racialized groups, would experience larger reductions in exposure to total and compositional PM_2.5_. The largest decreases were associated with nitrates, sulfates, and ammonia from agriculture, nitrates and black carbon from transportation, and organic matter from residential wood combustion. These findings have important implications for PM_2.5_ mitigation strategies aimed at reducing population exposure and associated disparities in Canada.

## Graphical abstract

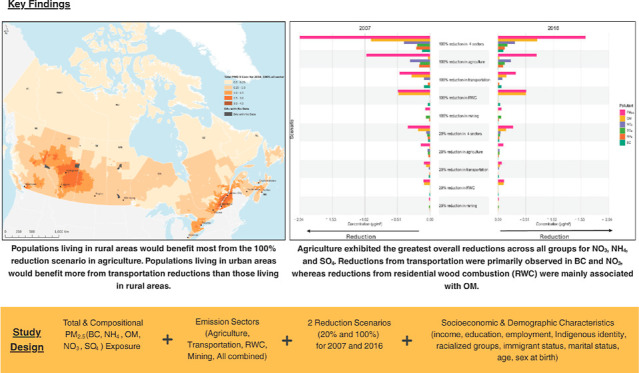

## Introduction

Globally, air pollution was responsible for an estimated 4.2 million premature deaths in 2019,^1^ most of which were attributed to particulate matter with an aerodynamic diameter under 2.5 μm (PM_2.5_).^2^ Due to its small size, PM_2.5_ can travel deep into the lungs and penetrate the bloodstream, inducing various health effects such as adverse birth outcomes, development of chronic diseases, and premature death.^3^ Formed by direct emissions and atmospheric chemical reactions, PM_2.5_ is derived from many different sources. In the high-income country of Canada, the major anthropogenic emission sources of PM_2.5_ include residential wood burning, on-road/off-road transportation, ore and mineral mining and manufacturing, and other sources (e.g., agriculture and forest fires), contributing to 33%, 14%, 20%, and 14%, respectively.^4^

While PM_2.5_ total mass has traditionally been a focus in air quality regulations, PM_2.5_ is inherently a mixture of various chemicals. The most commonly studied PM_2.5_ components in exposure modeling or epidemiology are sulfate (SO_4_^2–^), black carbon (BC), ammonium (NH_4_^+^), nitrate (NO_3_^–^), organic matter (OM), sea salt (SS), and mineral dust (Dust).^5–14^ A growing body of literature has linked individual PM_2.5_ chemical components to an array of adverse health outcomes (e.g., respiratory and cardiopulmonary mortality and reproductive outcomes).^5–14^

Recent studies have also documented that population exposure to PM_2.5_ is heterogeneous, often varying by people’s socioeconomic status or demographics, as seen in North America,^15–18^ Europe,^19–21^ Asia (e.g., China),^22,23^ and other parts of the world.^24^ For example, in a U.S. study, disproportionally greater exposure was reported among racialized subgroups with a lower income per household (≤US $25,000) between 2000 and 2010.^15^ Low-income and racialized subgroups were similarly found to be exposed to higher levels of PM_2.5_ in several other U.S. studies.^16,17^ In Canada, communities with lower income, higher unemployment, lower educational attainment, greater shelter-cost burden, or higher proportion of Indigenous peoples and racialized populations were reported to experience greater PM_2.5_ exposure between 2001 and 2016.^18^ These findings were supported by studies from Western Europe^20^ and China.^22^

While highlighting the social disparity in exposure to PM_2.5_ is important, it is equally important to show how air quality improvements may contribute to reducing exposure to total and compositional PM_2.5_, especially by socioeconomic and demographic dimensions. To date, such evidence remains sparse. Even less is known about how these potential benefits may vary across source-specific emission reductions. Thus, we conducted a nationwide study to quantify the expected reductions in total and compositional PM_2.5_ exposure from multiple emission sectors among different socioeconomic and demographic subgroups in Canada.

## Methodology

### Source-Specific Policy Scenarios

In light of Canada’s 2030 Emissions Reduction Plan (ERP) and its legislated objective of achieving net-zero emissions by 2050, which includes sector-specific measures to reduce both greenhouse gases and air pollutant from key sectors,^25–27^ we constructed two hypothetical emission-reduction scenarios to examine the potential range of population exposure benefits. These scenarios were not intended to represent specific policy proposals, but rather to provide sensitivity bounds for evaluating the magnitude and distributions of exposure reductions.

We modeled a zero-out (100% emission reduction), representing a theoretical upper-bound case to quantify the maximum achievable reductions in sector-attributable PM_2.5_ exposure, and an incremental scenario (20% emission reduction), representing a more modest and policy-relevant reductions consistent with phased regulatory tightening or technological improvements. The zero-out scenario should therefore be interpreted as a counterfactual benchmark rather than a plausible short-term policy pathway.

The four sectors, agriculture, transportation, residential wood combustion (RWC), and mining, were selected due to their substantial contributions to ambient PM_2.5_ and their inclusion in federal mitigation and regulatory frameworks under the ERP and the Clean Air Regulatory Agenda (CARA).^25–27^ Under each scenario, we quantified changes in total PM_2.5_ mass and seven chemical components, expressed on both absolute (in μg/m^3^) and relative (in percentages) scales in 2007 and 2016 (detailed description below).

### Study Population and Data Source

Our study covered ten Canadian provinces, comprising approximately 97% of the Canadian population. An array of important socioeconomic and demographic characteristics were collected from Canadian censuses conducted by Statistics Canada every five years.^28^ We extracted 2006 and 2016 census profile data at the dissemination area (DA) level, which is the smallest geographical unit (with an average population of 400 to 700 persons) for which census information is publicly disseminated.

We conducted an urban versus rural sub-analysis for each socioeconomic and demographic covariate. DAs were classified as urban if they were located within a census metropolitan area or a census agglomeration, while the remainder of the DAs were classified as rural.

### Exposure Assessment

We considered changes in PM_2.5_ total mass and seven major components—BC, NH_4_^+^, OM, NO_3_^–^, SO_4_^2–^, Dust, and SS—under the two aforementioned policy scenarios. Annual estimates of natural course (i.e., reference scenario), PM_2.5_ total mass (version V5.NA.05), and composition (version V5.NA.05.02) concentration at approximately 1 km^2^ resolution were developed by the Washington University Atmospheric Compositional Analysis Group by combining satellite-based aerosol optical depth retrievals with simulations from the GEOS-Chem chemical transport model (CTM) and calibrating to ground-level measurements using geographically weighted regression.^29^ This hybrid approach has been shown to provide PM_2.5_ total mass and components that are significantly correlated with cross-validated ground-based observations, for example: PM_2.5_ (*R* ^2^ = 0.43), SO_4_^2–^ (*R* ^2^ = 0.88), NO_3_^–^ (*R* ^2^ = 0.85), NH_4_^+^ (*R* ^2^ = 0.78), BC (*R* ^2^ = 0.67), and OM (*R* ^2^ = 0.63).^29^ We excluded Dust and SS because they are not expected to be influenced by emission reductions from the sectors examined.

To derive the impact of each hypothetical scenario, we applied the scenario-specific relative changes in simulated total and component mass concentrations to the corresponding hybrid values. The related GEOS-Chem simulations implemented source-specific emissions from the Canadian Air Pollutant Emissions Inventory^30^ for 2000–2016 to represent Canadian anthropogenic emissions and used the United States Environmental Protection Agency National Emission Inventory for 2011 ^31^ for American anthropogenic emissions. On-road/off-road transportation emissions data were available for all years between 2000 and 2016. Ore and minerals mining emissions data were available for the years 2000, 2005, 2010, and 2016 from Environment and Climate Change Canada, while linear interpolation was used to determine the intermediate years. Simulations were run at approximately 50 km^2^ spatial resolution and linearly interpolated for application to the hybrid concentrations used to represent annual changes in total and compositional PM_2.5_ mass across Canada in 2007 and 2016.

To obtain estimates of the simulated percent change in annual mean total and compositional PM_2.5_ concentration for all DAs, we first spatially extracted these exposure surfaces to the centroid of each dissemination block (DB) for 2007 and 2016. DBs are the smallest geographic area in Canada that contains population counts from the Census of Population, formed by the most recent road network information (equivalent to street blocks). These DB centroid concentration values were then used to calculate population-weighted concentrations at DAs.

### Socioeconomic and Demographic Covariates

For each DA, we obtained data *a priori* from the 2006 and 2016 Canadian censuses on: income expressed as low-income cutoffs after tax (LICO-AT), educational attainment, employment, Indigenous identity, visible minority expressed as racialized group, immigrant status, marital status, age, and biological sex at birth.

The income variables were derived from the LICO-AT variable and categorized as “low-income” based on the LICO-AT count, and the remainder of the population was categorized as “high-income”. The education variables were categorized as low education level (e.g., partially or fully completed high school and/or below bachelor degree) and high education level (e.g., college diploma or university degree (bachelor or higher)). The employment variables were categorized as employed and unemployed population. Indigenous identity variable was categorized as those who identified as Indigenous peoples (i.e., First Nations, Inuit, or Métis) and those who did not. The racialized group variable was derived from the visible minority variable and categorized as those who were from a racialized group and those who were not (i.e., white). The immigrant status variable was categorized as nonimmigrants and immigrants (e.g., landed immigrants, permanent residents, or naturalized citizens). The marital status variable was categorized as a single status (e.g., single, widowed, separated, or divorced) and a married status (e.g., married or common-law). The age variable was categorized as seniors (≥60 years) and non-seniors (<60 years). The sex at birth variable was categorized as the population that was either male or female.

The annual average population-weighted PM_2.5_ reductions (total and compositional) were calculated by using linked census variable data at the DA level for each subgroup and policy scenario for 2007 and 2016.

### Statistical Analyses

We calculated descriptive statistics for absolute concentration reductions (100% versus 20%) in population-weighted total mass and compositional PM_2.5._ This was done for 2007 and 2016 by four major emission sectors for the entire population and for specific subgroups according to the aforementioned socio-economic and demographic covariates. To visually represent the trends, we used bar charts (i.e., percentage and concentration reductions) and Sankey plots created for total mass PM_2.5_ and its five chemical components in each scenario and year using mean values. To compare differences in total and compositional PM_2.5_ exposure among selected subgroups, we performed Welch’s *t*-test (with Satterthwaite approximation) under the assumption of unequal variances between the two groups being compared (significance at the 0.05 level).

Our regional analysis was conducted for four Canadian regions (i.e., Western, Prairie, Central, and Atlantic). The Western region included the province of British Columbia. The Prairies included Alberta, Manitoba, and Saskatchewan. The Central region covered Ontario and Quebec. Lastly, the Atlantic region covered Nova Scotia, New Brunswick, Prince Edward Island, and Newfoundland and Labrador. Additional consideration was on urban versus rural residential neighborhoods to evaluate source-specific exposure.

We used SAS Enterprise Guide version 7.1 ^32^ for data management and descriptive statistical analysis, R version 4.4.0 ^33^ for constructing the plots, and ArcGIS version 10.6 ^34^ to create maps.

## Results

The ten Canadian provinces included in this study were home to 31.6 million people in 2006, which further increased to 34.5 million by 2016 (Table S1). The majority of the study population were under 60 years old (referred to as “non-seniors”), born in Canada (referred to as “non-immigrants”), and employed, held a college diploma or university degree, and lived in households with higher after-tax annual income (Table S1).

### Nationwide Exposure Reductions under the 100% Scenario

We observed that in 2007, the population-weighted average of annual mean exposure to total PM_2.5_ across the ten provinces was 7.66 μg/m^3^, decreasing by 5.89 μg/m^3^ in 2016. Among the major components of PM_2.5_, the population-weighted annual means in 2007 was 4.54 μg/m^3^ (59% of total PM_2.5_) for OM, 1.57 μg/m^3^ (20%) for SO_4_^2–^, 0.72 μg/m^3^ (9%) for NO_3_^–^, 0.65 μg/m^3^ (8%) for NH_4_^+^, and 0.48 μg/m^3^ (6%) for BC. Similar compositional patterns were observed in 2016.

If emissions from the four selected sectors were completely eliminated across Canada, average annual concentrations of total PM_2.5_ would decline from 7.66 μg/m^3^ by 2.04 μg/m^3^ in 2007 and from 5.89 μg/m^3^ by 1.61 μg/m^3^ in 2016—representing a 26% reduction in both years in this country (Figures 1 and S1). Based on the current hypothetical scenario, the largest reductions in PM_2.5_ and its components came from eliminating agriculture emissions, followed by transportation, RWC, and mining (Figure 1). For example, in 2007, eliminating emissions from agriculture alone would result in significant decreases in NO_3_^–^ (40%), NH_4_^+^ (28%), and SO_4_^2–^ (11%), leading to an overall reduction of 13% in total PM_2.5_ (Figure 1; Table S2). A similar pattern was seen in 2016. For the transportation sector, the greatest reductions in 2007 were observed for BC (17%), NO_3_^–^ (13%), OM (7%), and NH_4_^+^ (5%), with comparable reductions in 2016 (Figure 1; Table S2).

**Figure 1:**
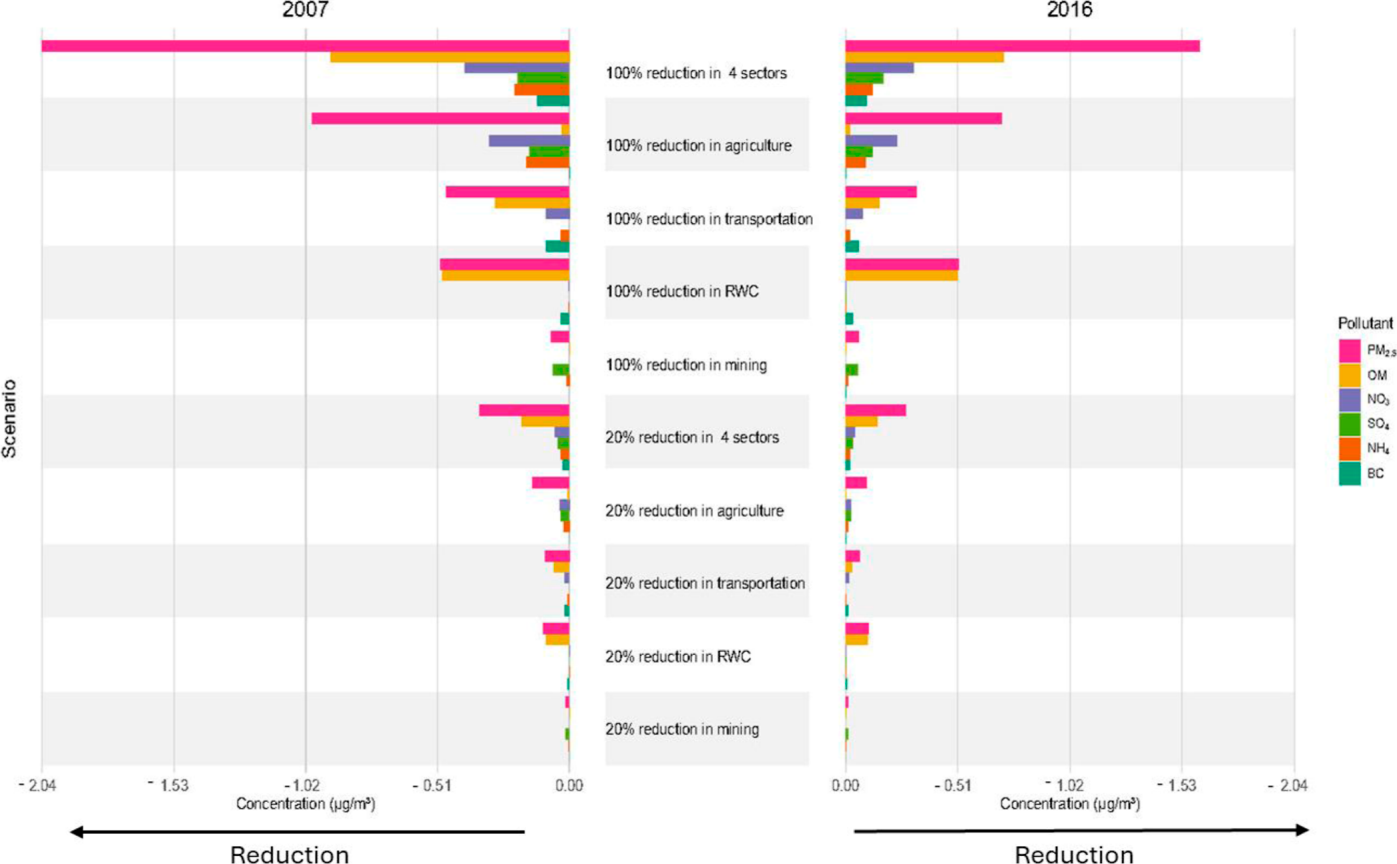
Population-weighted reductions in total and component PM_2.5_ concentrations (μg/m^3^) under 100% and 20% emission reductions from agriculture, transportation, residential wood combustion (RWC), and mining in 2007 and 2016, shown individually and combined.

When all four sectors were considered together, the expected exposure reductions would be 53% (0.40 μg/m^3^) for NO_3_^–^, 35% (0.21 μg/m^3^) for NH_4_^+^, 23% (0.12 μg/m^3^) for BC, and 22% (0.92 μg/m^3^) for OM in 2007. In 2016, the expected reductions would be 44% (0.31 μg/m^3^) for NO_3_^–^, 35% (0.12 μg/m^3^) for NH_4_^+^, 30% (0.12 μg/m^3^) for BC, and 24% (0.72 μg/m^3^) for OM (Table S2). Additionally, SO_4_^2–^ concentrations would decrease by 14% (0.20 μg/m^3^) in 2007 and by 20% (0.17 μg/m^3^) in 2016 (Table S2).

### Subgroup-Specific Exposure Reductions under the 100% Scenario

The expected reductions in exposure to PM_2.5_ and its major components were found to be heterogeneous across socioeconomic statuses and demographics (Tables S3–S11), particularly when emissions from the agriculture, transportation, and RWC sectors were mitigated. When these three sectors were combined, we found that groups experiencing socioeconomic disadvantage would benefit the most if the 100% emission reduction scenario was implemented between 2007 and 2016 (Tables S3–S11). For example, individuals with low-income (Figure 2; Table S3), immigrants (Figure 4, Table S5), and racialized groups (Figure 5; Table S6) consistently saw the largest benefits from emission reductions in these three sources (total PM_2.5_ reductions varied between ∼13 and 7% (equivalent to 1.18–0.51 μg/m^3^) for racialized groups and ∼12 and 6% (1.04–0.52 μg/m^3^) for low-income groups and immigrants). These results suggest that groups experiencing socioeconomic disadvantage are currently living in neighborhoods with higher PM_2.5_ exposure from agriculture, transportation, and RWC sectors. In addition, younger individuals (i.e., non-seniors) (Figure 3; Table S4), singles (Table S9), non-Indigenous (Table S10) and employed (Table S8) people would experience significant benefits from reductions. On the other hand, higher-income groups, non-immigrants, white, individuals over 60 years of age (i.e., seniors), married/common-law, and unemployed populations would experience more modest gains from reductions between 2007 and 2016. Surprisingly, in 2007, PM_2.5_ reductions were consistently larger for people with higher education levels than for those with lower education levels (Table S7). Specifically, in the agriculture sector, the high-education groups experinced reductions of 1.09 μg/m^3^ compared to1.02 μg/m^3^ for the low-education group lower education attaintment In transportation, reductions were 0.55 μg/m^3^ versus 0.48 μg/m^3^, and in RWC, 0.54 μg/m^3^ versus 0.50 μg/m^3^ for the high- and low-education groups, respectively (Table S7). A similar pattern was observed in 2016, with the high-education group continuing to experience slightly greater reductions overall. Regarding the sex difference, there was no significant difference between males and females exposed to PM_2.5_ and its chemical components across all emission sectors for 2007 and 2016 (Table S11). Lastly, there were consistently no significant differences in PM_2.5_ reductions between groups of all socioeconomic and demographic characteristics for emissions coming from mining between 2007 and 2016.

**Figure 2:**
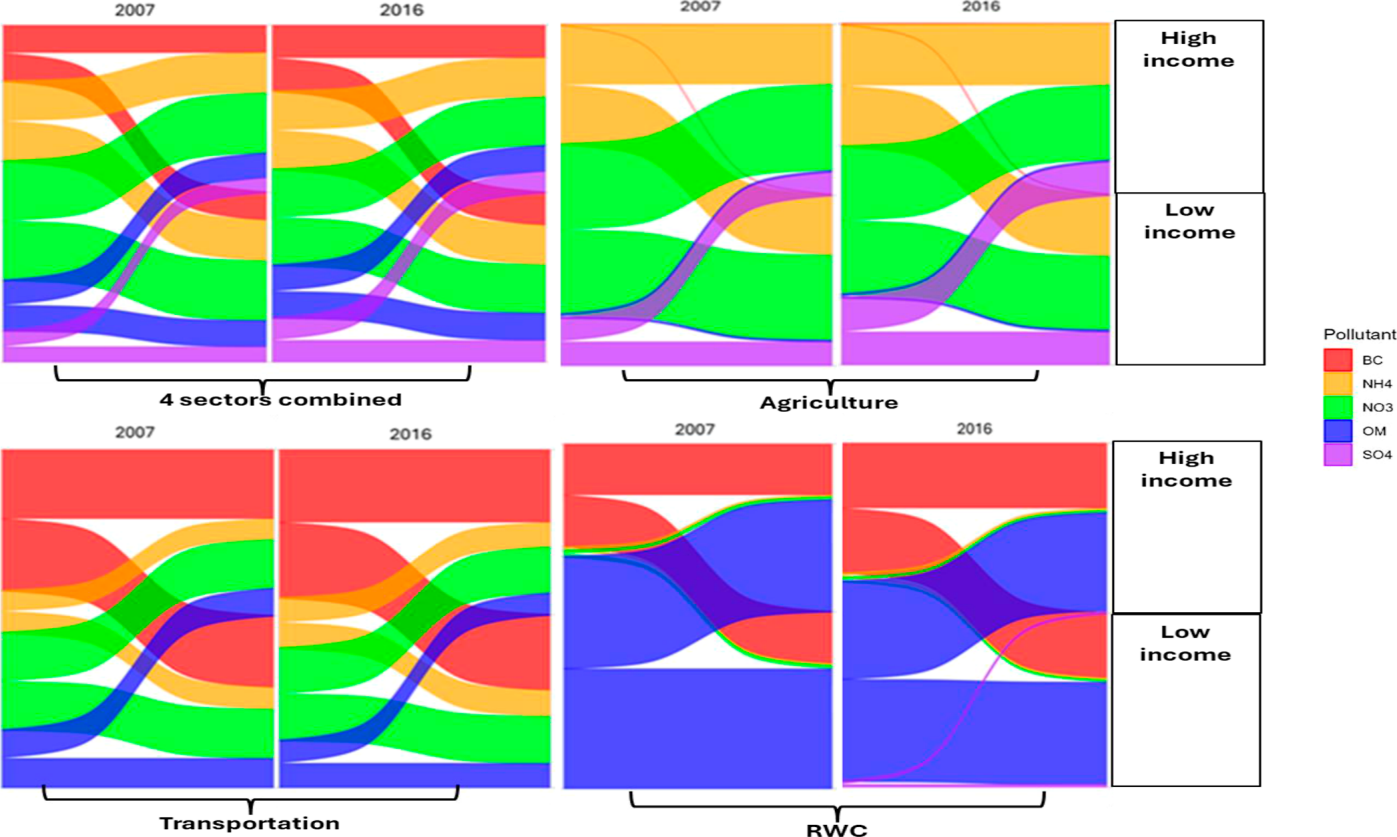
Absolute reductions in compositional PM_2.5_ concentrations (μg/m^3^) under 100% emissions removal from individual sectors (agriculture, transportation, and residential wood combustion (RWC)) and all sectors combined in 2007 and 2016, shown for groups with low-income versus groups with high-income per household.

**Figure 3:**
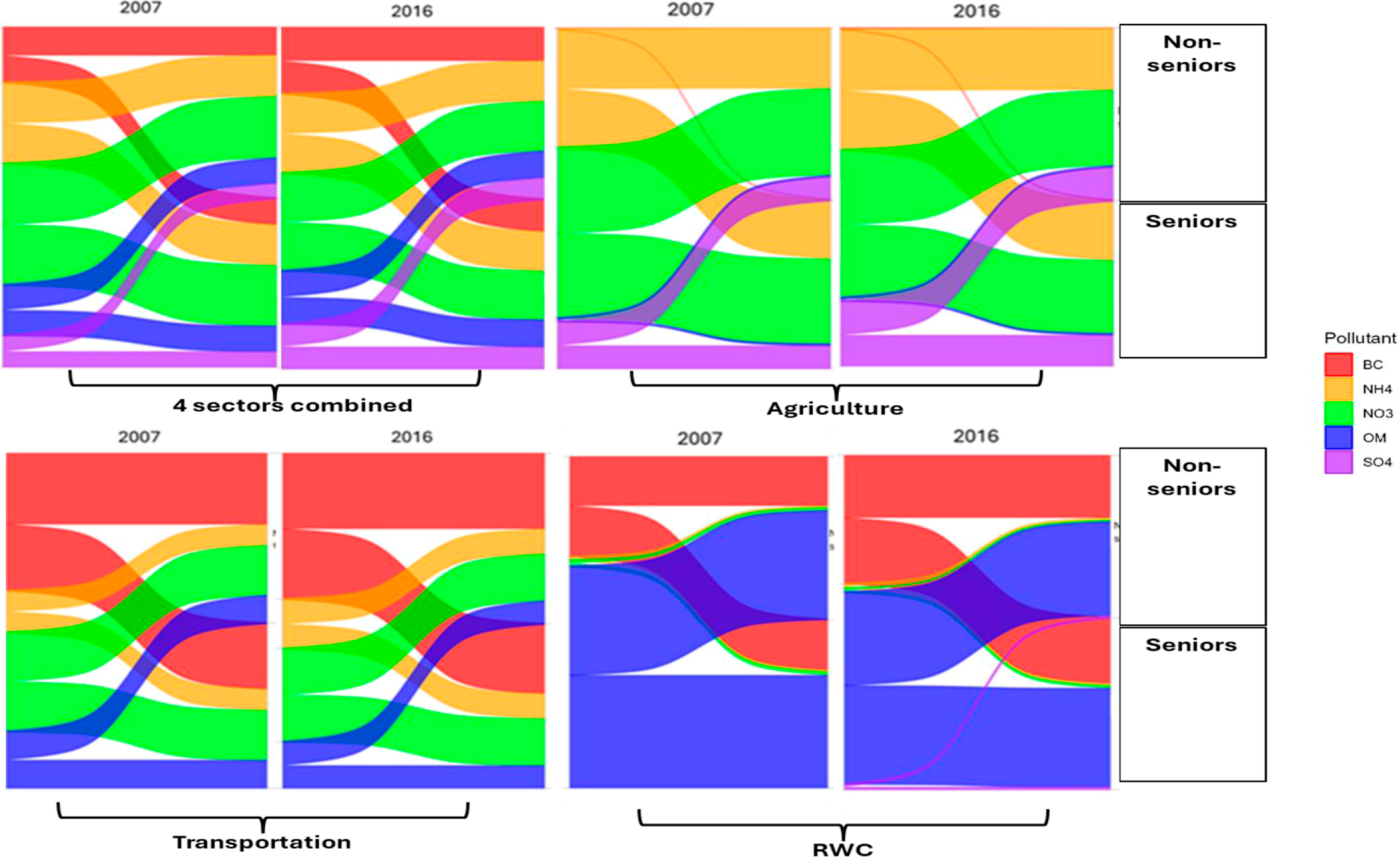
Absolute reductions in compositional PM_2.5_ concentrations (μg/m^3^) under 100% emissions removal from individual sectors (agriculture, transportation, and residential wood combustion (RWC)) and all sectors combined in 2007 and 2016, shown for seniors (≥60 years) and non-seniors (<60 years).

**Figure 4:**
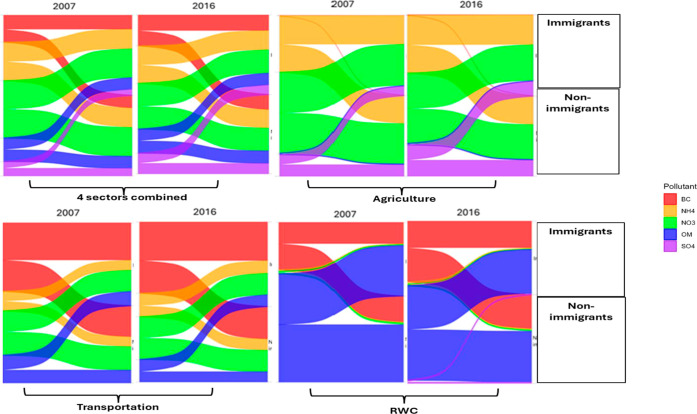
Absolute reductions in compositional PM_2.5_ concentrations (μg/m^3^) under 100% emissions removal from individual sectors (agriculture, transportation, and residential wood combustion (RWC)) and all sectors combined in 2007 and 2016, shown for immigrants and non-immigrants.

**Figure 5:**
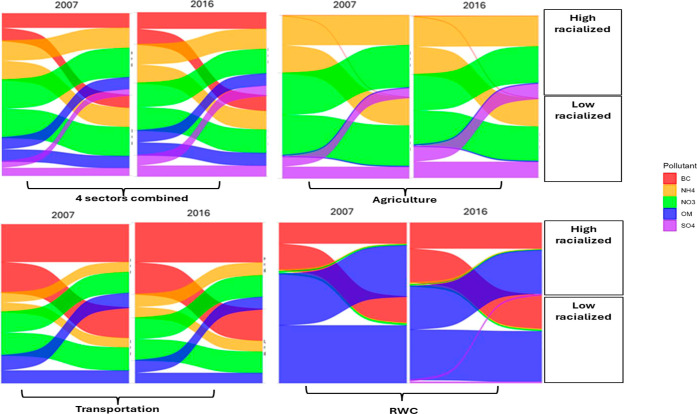
Absolute reductions in compositional PM_2.5_ concentrations (μg/m^3^) under 100% emissions removal from individual sectors (agriculture, transportation, and residential wood combustion (RWC)) and all sectors combined in 2007 and 2016, shown for populations living in areas with high and low proportions of racialized residents.

Across the population subgroups, the rank order of sector-specific impacts was consistent. Among individual sectors, mitigating emissions from agriculture would produce the largest overall reductions, followed by transportation and RWC. For example, low-income people experienced slightly higher reductions in PM_2.5_ under the 100% reduction scenario with 1.08 μg/m^3^ (agriculture), 0.55 μg/m^3^ (transportation), and 0.58 μg/m^3^ (RWC) compared to high-income people with 1.04 μg/m^3^ (agriculture) and 0.49 μg/m^3^ (transportation), and 0.51 μg/m^3^ (RWC) (Table S3). Mining produced the smallest gains (by 0.07 μg/m^3^ in 2007 and by 0.01 μg/m^3^ in 2016) in exposure reductions. Similar patterns would be observed in racialized groups and other subgroups.

The overall reductions in specific components also varied among the subgroups. In agriculture, the biggest component reductions were in NO_3_^–^, NH_4_^+^, and SO_4_^2–^, with the largest overall decreases of 40% (equivalent to 0.35 μg/m^3^), 28% (0.18 μg/m^3^), and 11% (0.17 μg/m^3^), respectively, for low-income groups (Table S3). High-income groups experienced similar yet statistically different reductions (Figure 2; Table S3). In transportation, largest reductions were for BC (19% or 0.12 μg/m^3^) and NO_3_^–^ (13% or 0.11 μg/m^3^) for low-income groups versus for BC (18% or 0.10 μg/m^3^) and NO_3_^–^ (13% or 0.09 μg/m^3^) for high-income groups (Figure 2; Table S3). In the RWC sector, the largest reduction was for OM (15% or 0.69 μg/m^3^) for low-income groups versus high-income groups (14% or 0.61 μg/m^3^) (Figure 2; Table S3). The mining sector also displayed chemical-specific reductions, yet this was smaller compared to the other sectors. These pollutant reduction patterns were consistent across emission sectors and other subgroups (e.g., immigrants and racialized groups) and considerable segments of the population (non-seniors, highly educated groups, employed, and singles).

In a stratified sub-analysis of urban and rural DAs, we observed a pattern of higher reductions in urban, rather than rural areas, across groups for both scenarios (20% and 100%) in 4 sectors (Figures S2–S4). The urban reductions were similar to the nation-wide reduction levels. These results support the idea that cities tend to have elevated PM_2.5_ levels in comparison to rural areas, which may contribute to why highly educated, single, and younger groups benefitted more from reductions as they often reside in urban areas.

### Exposure Reductions under the 20% Scenario

If emissions from all four sectors were reduced by 20% (as opposed to 100%), low-income groups, immigrants, racialized groups, singles, educated, non-Indigenous, and employed individuals would similarly experience tangible reductions in exposure to total and compositional PM_2.5_ between 2007 and 2016, albeit to a lower degree (Tables S3–S11). On the other hand, no significant difference would be observed between seniors and non-seniors (Table S4), or between males and females (Table S11). Among the chemical components of PM_2.5_, the greatest decreases would be seen for NO_3_^–^ (∼7%) and NH_4_^+^ (∼5%).

For individual sectors, significant differences in exposure reductions would be observed among certain groups, with the magnitudes of these differences varying by PM_2.5_ components, often very small. For example, in 2007, immigrants experienced reductions in agriculture-related total PM_2.5_ exposure by 0.17 μg/m^3^ (1.88%) versus 0.14 μg/m^3^ (1.82%) for non-immigrants (Table S5). Of PM_2.5_ components, the greatest decreases would be associated with NO_3_^–^, SO_4_^2–^, and NH_4_^+^ emitted from agriculture in 2007, although this was not always significant between the groups with other socioeconomic and demographic characteristics. For PM_2.5_ emitted from the transportation sector, low-income groups, immigrants, racialized groups, highly educated, employed, and non-Indigenous people also experienced significant reductions in 2007. For PM_2.5_ emitted from the RWC sector, low-income groups, non-seniors, non-Indigenous people, singles, employed, and highly educated groups experienced reductions in 2007. In contrast, for mining, no significant subgroup differences were observed in 2007, except for SO_4_^2–^ and NH_4_^+^ between non-seniors and seniors (Table S4) and racialized groups (Table S6). Overall differences between subgroups were generally small, particularly in 2016, meaning that 20% reductions in pollutants would make minimal differences across socioeconomic and demographic groups.

### Geographical Patterns

Beyond socioeconomic and demographic factors, reductions in total and compositional PM_2.5_ concentrations also varied geographically. The largest benefits from the zero-out scenario were found in Ontario, Quebec, Alberta, and British Columbia (Figures S5–S16). In Ontario, reductions of PM_2.5_, NO_3_^–^, NH_4_^+^, OM, and BC were concentrated in southern regions, especially around large cities, including Windsor, London, Hamilton, Greater Toronto Area, and Ottawa, but less so in central and northern areas. In Quebec, significant reductions occurred in Montreal, Quebec City, and other southern regions. but not in central/northern Quebec. In British Columbia, reductions of PM_2.5_ and OM were most notable in central and southern areas, particularly around Vancouver. In Alberta, reductions of PM_2.5_, NO_3_^–^, and NH_4_^+^ were most evident in central and southern parts, as well as around large cities, such as Edmonton and Calgary. Residents of these places experienced reductions in 2007, though not consistently in 2016. For SO_4_^2–^, no geographic patterns were found (Figures S7 and S13).

## Discussion

To the best of our knowledge, this is the first nationwide Canadian study to examine how socioeconomic and demographic subgroups benefit from exposure reductions in both total and compositional PM_2.5_ under different policy scenarios. Our findings also quantify how emission reductions in major sectors can contribute to lowering PM_2.5_ levels by population subgroups—an important step toward advancing environmental justice. We found that if emission reductions had been implemented on major sectors, there would have been a tangible decline in total and compositional PM_2.5_ exposure across the country and subgroups with varying socioeconomic and demographic characteristics. Despite improvements in mitigating PM_2.5_ over the last several decades, it is crucial to recognize that emission reductions would not have benefitted the Canadians equally. Particularly, emissions from agriculture, transportation, and RWC sectors contributed disproportionately to equity-seeking communities, such as those with low-income, immigrants, and racialized groups, who would have experienced larger reductions in exposure to both total and compositional PM_2.5_ (e.g., NO_3_^–^, SO_4_^2–^, NH_4_^+^, BC, and OM). Notably, these patterns persisted under both 100% and 20% emission reduction scenarios, suggesting that even moderate reductions would lead to declines in PM_2.5_ exposure for equity-seeking populations in Canada. We also found that if all emission reductions had been fully and realistically implemented, residents with distinct socioeconomic and demographic characteristics (e.g., people ≤ 60 years, employed, with single marital status, and highly educated groups) would have experienced the greatest benefits. This is especially important due to growing evidence that even relatively low PM_2.5_ levels can have significant health impacts.^35–38^ In 2016, Canada’s population was approximately 35 million, with an estimated 10,000 premature deaths attributable to PM_2.5_ exposure.^39^ As Braveman and Gruskin defined, health is not just the presence of disease but also a state of both physical and mental well-being.^40^ Populations experiencing socioeconomic disadvantage are particularly vulnerable to environmental exposures, which may exacerbate existing health inequities.^40^ Given that PM_2.5_ levels in Canada are among the lowest globally,^4,36,37^ our estimated reductions in exposure may have important implications for addressing health disparities in Canada and elsewhere.

A growing body of literature has shown social disparities in observed exposure to PM_2.5_ in North America,^15–18,41^ Europe,^19–21^ and China^22,23^ and across the globe.^24^ For example, an American study found that racialized communities (i.e., non-Hispanic Black, Hispanic, and non-Hispanic Asian) and groups with lower income were consistently exposed to higher levels of PM_2.5_, with these disparities persisting over time.^15^ In a nationwide Canadian study, racialized groups, people with lower-income households (<30,000$), seniors (66+ years), and immigrants were exposed to higher levels of PM_2.5_, especially in parts of southern Ontario (particularly around Toronto and Windsor) and in major urban centers.^41^ Similarly, in Western Europe, racial/ethnic minority communities were shown to experience greater exposure to PM_2.5_,^20^ specifically Turkish, Moroccan, Surinamese, and Indian ethnic groups.^21^ Furthermore, in a study conducted in China, low-income communities were found to be exposed to higher PM_2.5_ than their wealthier counterparts.^22^ Although, these previous studies highlighted the existing exposure disparities, they did not consider how these communities may be differentially affected by reductions in major emission sources. In our study, we demonstrated that groups with lower-income, immigrants, and racialized groups would experience more reductions in PM_2.5_ exposure from agriculture, transportation, and RWC emission sources in Canada. We observed further disparities between lower-income and higher-income groups when stratifying for urban and rural areas for both 2007 and 2016 from the 100% reduction in 4 sectors scenario. Across all pollutants, excluding SO_4^2−^_, the reductions for both groups were similar in urban areas, but there is a notable difference among rural areas. Lower-income groups in rural areas experienced the smallest reductions, specifically from NH_4_^+^ and overall PM_2.5_ (Figure 6). Among immigrants, higher educated groups, and racialized groups, we observed similar patterns of higher reductions in urban areas that were close to the national average, while the rural reductions were more modest in comparison (Figures S2–S4).

**Figure 6:**
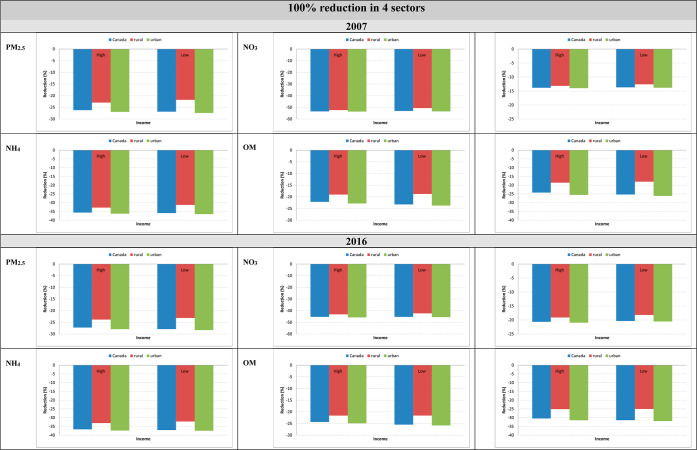
Estimated percent reduction in PM_2.5_ components following complete emission removal from four sectors combined in Canada (2007 versus 2016): differences across high- and low-income groups and urban–rural populations.

Apart from the established equity-deserving communities (e.g., low-income groups), we also found that subgroups of people that were younger and employed could benefit more from reductions in total and compositional PM_2.5_ concentrations emitted from the agriculture, transportation, and RWC sectors. Although these groups are not socially disadvantaged, they are significant segments of population (e.g., younger individuals comprised between 86% (in 2007) and 84% (in 2016) versus employed individuals comprised between 93% (in 2007) and 79% (in 2016) of the Canadian population) (Table 1S). These individuals tend to live in urban rather than rural areas, where PM_2.5_ and other pollutants are often more elevated,^42^ depending on the local emission source, as depicted in the urban and rural analysis. An American study found that communities with a greater percentage of unemployed people and those under 64 years of age were at a larger exposure disparity.^43^ In The Netherlands, they found that younger people had higher PM_2.5_ exposure, especially those living in urban areas with increased traffic-related emissions.^21^ In Canada, a difference in PM_2.5_ exposure was observed for younger people living in urban centers compared to rural areas.^41^ Globally, a review by Yu et al. found that PM_2.5_ sources differ between rural and urban areas: urban residents often face higher absolute and relative PM_2.5_ exposure from population density, industry, traffic, and construction, while rural residents occasionally experience elevated levels from agriculture and biomass burning events.^44^

Interestingly, we also found that groups with higher educational levels would experience greater benefits from emission reductions in comparison to those with lower educational levels. This is contrary to previous studies that have shown that people with lower educational attainment were more likely to be exposed to higher PM_2.5_ levels.^42,43,45,46^ This divergency might be related to immigration since the majority of immigrants are highly educated due to it being a key selection criteria in Canada’s immigration system.^47^ For example, in 2021, recent immigrants (55%) and those who immigrated over ten years ago (40%) had at least a bachelor’s degree or higher.^47^ Moreover, it may be due to our categorization of lower education and higher education, which may differ from existing studies, or because more educated people tend to live in urban areas where air pollution is often higher.^18,41^

We initially found that non-Indigenous people would benefit more from reductions than Indigenous peoples when looking at the national average; however, the stratified sub-analysis of urban and rural DAs revealed more nuanced differences between the urban and rural Indigenous populations. We observed that Indigenous people residing in urban areas would benefit more from reductions than those in rural areas or on the national average (Figure 7). These urban reductions for Indigenous peoples were much more similar to the non-Indigenous peoples reductions for both Canada-wide and urban areas. Although the stratified sub-analysis highlighted the large differences between the urban and rural Indigenous populations, it is important to note the limited representation of Indigenous populations as this study focused on the 10 provinces, and thus, excluded all populations located in Northwest Territories, Nunavut, and Yukon. This requires further research in order to capture the populations living in the territories.

**Figure 7:**
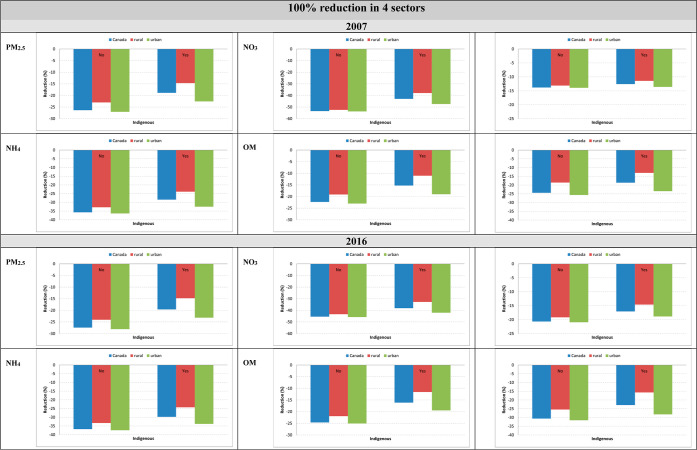
Estimated percent reduction in PM_2.5_ components following complete emission removal from four sectors combined in Canada (2007 versus 2016): differences across Indigenous and non-Indigenous groups and urban–rural populations.

Lastly, a difference in the marital status also emerged. We found that people not in a married or common-law union experienced greater benefits from emission reductions than those who were married or in common-law partnerships. This may be due to individuals with single marital status residing in urban areas or more likely to be younger. This finding suggests that marital status may not be a strong modifier of exposure, a pattern that warrants further investigation.

Since individuals are simultaneously exposed to various chemical components of PM_2.5_ (and other pollutants) in a complex mixture, there is growing interest in studying compositional PM_2.5_ in addition to total mass. The previous literature shows that exposure to different PM_2.5_ constituents and emission sources is linked to a range of health outcomes, from respiratory and cardiovascular diseases to reproductive effects.^5–12,14^ For example, the California Teachers Study found strong associations between exposure to NO_3_^–^ and SO_4_^2–^ and cardiovascular and respiratory mortalities.^5^ Another US Medicare study found positive associations between exposure to elemental carbon and NO_3_^–^ and mortality, but an inverse association between SO_4_^2–^ and mortality was found.^10^ Another American study, with over 13 million Medicare participants, found that exposure to BC, NO_3_^–^, OM, and SO_4_^2–^ was significantly associated with all-cause mortality.^48^ In a Danish cohort study, an association between exposure to secondary organic aerosols and mortality was found.^13^ These findings suggest that different PM_2.5_ components carry varying degrees of health risk.

In this Canadian study, we found that agriculture produced the largest overall reductions in levels of NO_3_^–^, NH_4_^+^, and SO_4_^2–^, while transportation delivered substantial but smaller improvements in levels of BC and NO_3_^–^, and RWC yielded measurable but comparatively smaller gains in levels of OM between 2007 and 2016. SO_4_^2–^, NO_3_^–^, and NH_4_^+^ mainly come from agricultural fertilizers, including potash and those that are nitrogen-rich, which are mainly used to stimulate growth and promote the health of many crops.^49,50^ The Prairies, Southern Ontario, and British Columbia are major producers of cereals, oilseeds, pulses, and forage crops (e.g., alfalfa and grasses), as well as major livestock industries driven by cattle, poultry, and hog production,^51^ which are the main producers of these pollutants, especially in rural areas. Transportation is another major contributor, especially diesel engines in off-road (7 kt or 32%) and on-road (2.2 kt or 10%) vehicles.^52^ Urban communities would experience more benefits in reductions in levels of BC emitted from transportation due to the high density of roadways and traffic in urban areas. Approximately 19% of Canadian households use wood-burning appliances as a source of heat, for cooking, or as a back-up during power-outages.^53^ The RWC sector is responsible for OM emissions, and based on a 2021 Statistics Canada survey, about 4% of rural households and about 1% of urban households use heating stove burning wood pellets or coal for cooking or heating purposes.^54^ Reducing emissions from these sources would particularly benefit low-income and racialized groups, as well as those who are single, highly educated, immigrants, non-seniors, and employed people, as these groups are more likely to live near major emission sources in British Columbia, Alberta, Ontario, and Quebec.

Our study has several strengths. First, it contributes to the growing literature on environmental justice in air pollution exposure by further highlighting which equity-deserving communities benefit the most from reductions in exposure from the emissions of four major sectors. Additionally, our study leveraged the novel estimates of spatially resolved total and compositional PM_2.5_ absolute concentrations simulated under different intervention scenarios between 2007 and 2016. These results provide more direct evidence in informing targeted policy interventions to decrease public burden from PM_2.5_ exposure, especially since PM_2.5_ is a major contributor to global disease burden worldwide.^1^ There is growing evidence that exposure to PM_2.5_ results in various adverse health outcomes, including cardiovascular, respiratory, neurological, reproductive, and developmental diseases, as well as cancers.^3,39,55–57^ In addition to the PM_2.5_ total mass, this study provided novel information on which specific PM_2.5_ components and emission sectors resulted in the largest overall reductions. This provides illuminating information to the previous literature that showed not all PM_2.5_ components are equally toxic and harmful to human health.^5,10,48^ Lastly, by considering the geographic variations in sector-specific reductions in emissions for PM_2.5_ and major constituents, we could determine which Canadian regions experience the greatest or smallest reductions in observed concentrations. All of these factors are pivotal for environmental justice and determining appropriate mitigation measures.

Our study is not without limitations. First, uncertainty in true exposure could be introduced by differences in individual-level exposure and the area-weighted means provided by the hybrid PM_2.5_ data set satellite, as well as uncertainties in the hybrid data set itself. CTMs tend to operate at relatively coarse grid scales, which can cause underestimation of exposure differences across small spatial scales.^58^ This limitation is particularly relevant when assessing within-city variability, where localized pollution gradients may disproportionately affect vulnerable populations. Second, our study described differences in exposure across socioeconomic and demographic groups; however, since we did not model associations between these variables and exposures, we cannot determine whether any specific socioeconomic and demographic factor independently contributes to higher exposure risk. Lastly, although we focused on four major emission sources, there are other emission sources (i.e., electric power generation, power plants, industrial facilities, oil and gas industry, and forest fires) that emit PM_2.5_.^4^ Further research is warranted to better evaluate public health benefits from sector- and component-specific PM_2.5_ reductions across small spatial scales.

## Supplementary Material



## Notes

Socioeconomic and demographic characteristics of Canadians from 2006 and 2016 Census of Population; variations in population-weighted mean absolute concentrations of total PM_2.5_ and its five chemical components by the emission sector and % reduction (in parentheses) for 100% and 20% scenarios between 2007 and 2016; bar graph representing 100% and 20% reductions in population-weighted percentage of total mass PM_2.5_ and its five major components for 2007 and 2016 by four individual sector (i.e., agriculture, transportation, residential wood combustion, and mining), as well as four sectors combined; estimated reduction in exposure of total mass PM_2.5_ and its five major components for low versus high-income per household after tax (LICO-AT) groups by four selected emission sectors and combined for 100% and 20% scenarios between 2007 and 2016; estimated reduction in exposure of total mass PM_2.5_ and its five major components for nonseniors (under 60 years of age) versus seniors (60+ years of age) groups by four selected emission sectors and combined for 100% and 20% scenarios between 2007 and 2016; estimated reduction in exposure of total mass PM_2.5_ and its five major components for immigrants versus nonimmigrants by birth groups by four selected emission sectors and combined for 100% and 20% scenarios between 2007 and 2016; estimated reduction in exposure of total mass PM_2.5_ and its five major components for racialized versus nonracialized groups by four selected emission sectors and combined for 100% and 20% scenarios between 2007 and 2016; estimated reduction in exposure of total mass PM_2.5_ and its five major components for groups of people with low (i.e., partially or fully completed high school) versus high (i.e., college diploma or university degree (bachelor or above)) educational attainment by four selected emission sectors and combined for 100% and 20% scenarios between 2007 and 2016; estimated reduction in exposure of total mass PM_2.5_ and its five major components for employed versus unemployed groups by four selected emission sectors and combined for 100% and 20% scenarios between 2007 and 2016; estimated reduction in exposure of total mass PM_2.5_ and its five major components for in marriage status (married or common-law) versus singles (never married, was widowed, divorced, or separated) groups by four selected emission sectors and combined for 100% and 20% scenarios between 2007 and 2016; estimated reduction in exposure of total mass PM_2.5_ and its five major components for Indigenous peoples (belonging to one or more of the following groups: Métis, Inuit, First Nations) versus non-Indigenous groups by four selected emission sectors and combined for 100% and 20% scenarios between 2007 and 2016; estimated reduction in exposure of total mass PM_2.5_ and its five major components for males versus females groups by four selected emission sectors and combined for 100% and 20% scenarios between 2007 and 2016; estimated percent reduction in PM_2.5_ components following complete emission removal from four sectors in Canada (2007 versus 2016): differences across immigrant status and urban–rural populations; estimated percent reduction in PM_2.5_ components following complete emission removal from four sectors in Canada (2007 versus 2016): differences across people socially categorized by race and urban–rural populations; estimated percent reduction in PM_2.5_ components following complete emission removal from four sectors in Canada (2007 versus 2016): differences across educational level and urban–rural populations; 100% policy reduction scenario of fine particulate matter (PM_2.5_) in four major emission sectors combined (i.e., agriculture, transportation, residential wood combustion, and mining) captured in Western (A), Prairies (B), Central (C), and Atlantic (D) regions during 2007; 100% policy reduction scenario of nitrate (NO_3_^–^) in four major emission sectors combined (i.e., agriculture, transportation, residential wood combustion, and mining) captured in Western (A), Prairies (B), Central (C), and Atlantic (D) regions during 2007; 100% policy reduction scenario of sulfate (SO_4_^2^)^−^ in four major emission sectors combined (i.e., agriculture, transportation, residential wood combustion, and mining) captured in Western (A), Prairies (B), Central (C), and Atlantic (D) regions during 2007; 100% policy reduction scenario of ammonium (NH_4_^+^) in four major emission sectors combined (i.e., agriculture, transportation, residential wood combustion, and mining) captured in Western (A), Prairies (B), Central (C), and Atlantic (D) regions during 2007; 100% policy reduction scenario of organic matter (OM) in four major emission sectors combined (i.e., agriculture, transportation, residential wood combustion, and mining) captured in Western (A), Prairies (B), Central (C), and Atlantic (D) regions during 2007; 100% policy reduction scenario of black carbon (BC) in four major emission sectors combined (i.e., agriculture, transportation, residential wood combustion, and mining) captured in Western (A), Prairies (B), Central (C), and Atlantic (D) regions during 2007; 100% policy reduction scenario of fine particulate matter (PM_2.5_) in four major emission sectors combined (i.e., agriculture, transportation, residential wood combustion, and mining) captured in Western (A), Prairies (B), Central (C), and Atlantic (D) regions during 2016; 100% policy reduction scenario of nitrate (NO_3_^–^) in four major emission sectors combined (i.e., agriculture, transportation, residential wood combustion, and mining) captured in Western (A), Prairies (B), Central (C), and Atlantic (D) regions during 2016; 100% policy reduction scenario of sulfate (SO_4_^2–^) in four major emission sectors combined (i.e., agriculture, transportation, residential wood combustion, and mining) captured in Western (A), Prairies (B), Central (C), and Atlantic (D) regions during 2016; 100% policy reduction scenario of ammonium (NH_4_^+^) in four major emission sectors combined (i.e., agriculture, transportation, residential wood combustion, and mining) captured Western (A), Prairies (B), Central Quebec (C), and Atlantic (D) regions during 2016; 100% policy reduction scenario of organic matter (OM) in four major emission sectors combined (i.e., agriculture, transportation, residential wood combustion, and mining) captured in Western (A), Prairies (B), Central (C), and Atlantic (D) regions during 2016; and 100% policy reduction scenario of black carbon (BC) in four major emission sectors combined (i.e., agriculture, transportation, residential wood combustion, and mining) captured in Western (A), Prairies (B), Central (C), and Atlantic (D) regions during 2016 (PDF)

## Abbreviations

BC: black carbon
CTM: chemical transfer model
DA: dissemination area
DB: dissemination block
Dust: mineral dust
kt: kiloton
NH_4_^+^: ammonium
NO_3_^–^: nitrate
OM: organic matter
RWC: residential wood combustion
SO_4_^2–^: sulfate
SS: sea salt
