# Supplementary material for: Differential Reductions in Total and Compositional PM2.5 Exposure across Socioeconomic and Demographic Groups from Emission Source Mitigation in Canada (2007–2016)
Source: Environ Sci Technol. 2026 Apr 14;60(16):12063–75. doi: 10.1021/acs.est.5c17598 (PMC13130957; doi:10.1021/acs.est.5c17598)
Supplement: Supplementary file 1 [file es5c17598_si_001.pdf]

Title: Differential Reductions in Total and Compositional $PM_{2.5}$ Exposure Across Socioeconomic and Demographic Groups from Emission Source Mitigation in Canada (2007-2016)

Authors: Anna Delic[1], Katarina Kunarac[1], Li Chen[1], Amanda Pappin[2], Aaron van Donkelaar[3], Randall Martin[3], Hong Chen[1,4,5,6]*

Affiliations: [1-]Environmental Health Science & Research Bureau, Health Canada, Ottawa, Ontario, K1A 0K9, Canada, [2-]Water and Air Quality Bureau, Health Canada, Ottawa, Ontario, K1A 0K9, Canada, [3-]Department of Energy, Environment & Chemical Engineering, Washington University, St. Louis, Missouri, 63112, USA, [4-]Public Health Ontario, Toronto, Ontario, M5G 1V2, Canada, [5-]ICES, Toronto, Ontario, M4N 3M5, Canada, [6-]Dalla Lana School of Public Health, University of Toronto, Toronto, Ontario, M5T 3M7, Canada

Summary: 34 pages (including cover sheet), 11 Tables and 16 Figures

Dissemination area (DA) is the smallest geography unit with a relatively stable geographical boundary. DAs are available for the entirety of Canada.[28] To calculate population-weighted DAs, the following formula was used:

"Population-weighted DA = $\sum$(population of DB*concentration of DB centroid) / $\sum$population of DA"

Table 1S. Socioeconomic and demographic characteristics of Canadians from 2006 and 2016 Census of Population.

Characteristics	2006 (%)	2016 (%)
Cohort size (N)	31,612,895	34,460,065
**Time-fixed covariates**		
Age		
Seniors (> 60 years)	4,335,255 (13.71)	5,479,905 (15.90)
Non-seniors ($\leq$ 60 years)	27,277,640 (86.29)	28,980,160 (84.10)
Sex at birth		
Female	16,136,925 (51.05)	17,488,485 (50.75)
Male	15,475,970 (48.95)	16,971,580 (49.25)
Marital status		
In status[a]	15,149,470 (52.91)	16,880,285 (57.66)
Single[b]	13,482,610 (47.08)	12,396,915 (42.34)
Immigrant status		
Immigrant[c]	6,186,950 (19.80)	7,540,830 (22.21)
Non-immigrant	24,788,720 (79.35)	26,412,615 (77.79)
Visible Minority		
Low minority rate	26,172,940 (83.78)	26,785,485 (77.73)
High minority rate	5,068,090 (16.22)	7,674,585 (22.27)
Employment status		
Employed	16,021,180 (93.44)	18,277,925 (78.91)
Unemployed	1,124,955 (6.56)	4,885,185 (21.09)
Education		
Low education[d]	6,840,250 (39.35)	6,664,385 (35.20)
High education[e]	10,541,865 (60.65)	12,267,005 (64.80)
Income[f]		
High income (> $115,000)	28,699,840 (91.61)	31,156,690 (90.74)
Low income (< $42,000)	2,629,980 (8.39)	3,179,260 (9.26)
Indigenous identity		
Non-indigenous	30,068,240 (96.00)	32,786,280 (95.14)
Indigenous Peoples[g]	1,172,790 (4.00)	1,673,780 (4.86)

Note: [a]-In status (married or common-law); [b]-Single (never married, separated, widowed, divorced); [c]-Immigrants (landed immigrants, permanent residents, or naturalized citizens); d-Low education level (partially or fully completed high school and/or below bachelor degree); e-High education level (college diploma or university degree (bachelor or higher)); f- Income (low versus high annual household income after-tax (LICO-AT)), g-Indigenous Peoples (First Nations, Métis, or Inuit).

Table 2S. Variations in population-weighted mean absolute concentrations of total $PM_{2.5}$ and its five chemical components by emission sector and % reduction (in parentheses) for 100% and 20% scenarios between 2007 and 2016.

		100% reduction (μg/m³)		20% reduction (μg/m³)	
Sector	Component	2007	2016	2007	2016
**All 4 sectors combined**	$PM_{2.5}$	2.04 (25.67%)	1.61 (26.49%)	0.35 (4.39%)	0.27 (4.49%)
	NO_3^-	0.40 (52.73%)	0.31 (44.32%)	0.06 (7.59%)	0.04 (6.18%)
	SO_4^{2-}	0.20 (13.70%)	0.17 (20.12%)	0.04 (2.92%)	0.03 (3.73%)
	NH_4^+	0.21 (35.04%)	0.12 (35.62%)	0.03 (5.37%)	0.02 (5.32%)
	OM	0.92 (21.92%)	0.72 (24.04%)	0.19 (4.39%)	0.14 (4.81%)
	BC	0.12 (23.48%)	0.12 (29.54%)	0.02 (4.70%)	0.02 (5.91%)
**Agriculture**	$PM_{2.5}$	1.00 (12.51%)	0.71 (11.75%)	0.14 (1.79%)	0.10 (1.60%)
	NO_3^-	0.31 (40.07%)	0.23 (32.84%)	0.04 (5.20%)	0.03 (3.92%)
	SO_4^{2-}	0.16 (11.18%)	0.12 (14.70%)	0.03 (2.41%)	0.03 (2.94%)
	NH_4^+	0.17 (27.71%)	0.09 (26.61%)	0.02 (3.96%)	0.01 (3.67%)
	OM	0.03 (0.81%)	0.02 (0.75%)	0.01 (0.15%)	0 (0.15%)
	BC	0 (0.12%)	0 (0.18%)	0 (0.01%)	0 (0.03%)
**Transportation**	$PM_{2.5}$	0.48 (6.04%)	0.32 (5.49%)	0.10 (1.21%)	0.06 (1.10%)
	NO_3^-	0.09 (12.69%)	0.08 (11.53%)	0.02 (2.54%)	0.02 (2.31%)
	SO_4^{2-}	0.02 (1.31%)	0.01 (1.08%)	0 (0.26%)	0 (0.22%)
	NH_4^+	0.03 (5.26%)	0.02 (5.96%)	0.01 (1.05%)	0 (1.19%)
	OM	0.29 (7.14%)	0.15 (5.60%)	0.06 (1.43%)	0.03 (1.12%)
	BC	0.09 (17.04%)	0.06 (17.46%)	0.02 (3.41%)	0.01 (3.49%)
**RWC**	$PM_{2.5}$	0.50 (6.21%)	0.51 (8.20%)	0.10 (1.24%)	0.10 (1.64%)
	NO_3^-	0 (0.39%)	0 (0.36%)	0 (0.08%)	0 (0.07%)
	SO_4^{2-}	0 (0%)	0 (0.51%)	0 (0%)	0 (0.10%)
	NH_4^+	0 (0.18%)	0 (0.41%)	0 (0.04%)	0 (0.08%)
	OM	0.61 (13.96%)	0.54 (17.66%)	0.12 (2.79%)	0.11 (3.53%)
	BC	0.03 (6.31%)	0.04 (11.86%)	0.01 (1.26%)	0.01 (2.37%)
**Mining**	$PM_{2.5}$	0.07 (0.92%)	0.06 (1.06%)	0.01 (0.18%)	0.01 (0.21%)
	NO_3^-	0 (0.41%)	0 (0.41%)	0 (0.08%)	0 (0.08%)
	SO_4^{2-}	0.06 (3.83%)	0.05 (5.98%)	0.01 (0.77%)	0.01 (1.20%)
	NH_4^+	0.01 (1.89%)	0.01 (2.65%)	0 (0.38%)	0 (0.53%)
	OM	0 (0.01%)	0 (0.03%)	0 (0%)	0 (0.01%)
	BC	0 (0.02%)	0 (0.04%)	0 (0%)	0 (0.01%)

Note: RWC: Residential wood combustion.

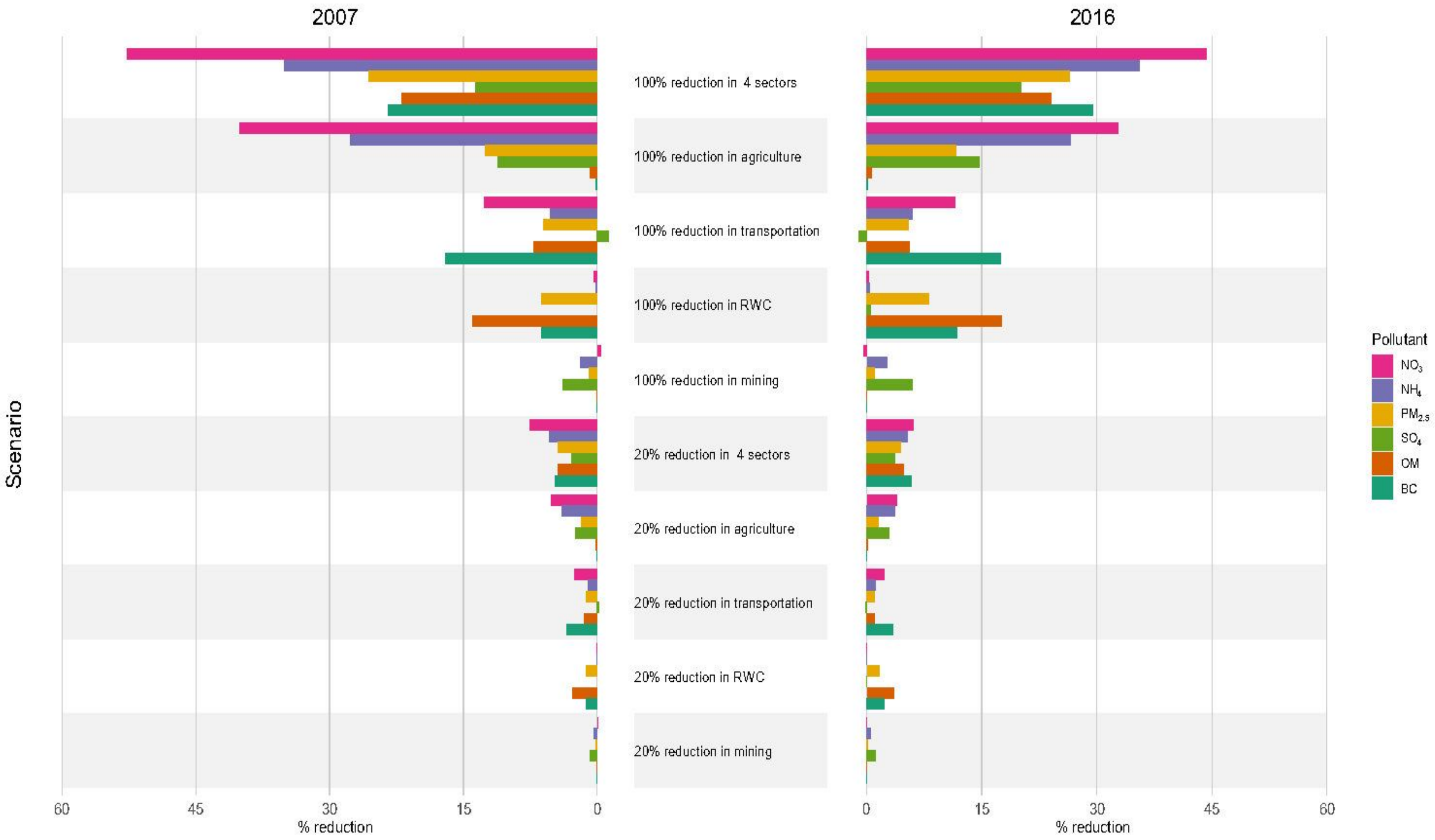

Figure 1S. Bar graph representing 100% and 20% reductions in population-weighted percentage of total mass $PM_{2.5}$ and its five major components for 2007 and 2016 by four individual (i.e., agriculture, transportation, residential wood combustion (RWC), and mining), as well as four sectors combined.

Table 3S. Estimated reduction in exposure of total mass $PM_{2.5}$ and its five major components for low versus high-income per household after tax (LICO-AT) groups by four selected emission sectors and combined for 100% and 20% scenarios between 2007 and 2016.

		100% reduction (μg/m³)						20% reduction (μg/m³)					
		High income	Low income	Sig.	High income	Low income	Sig.	High income	Low income	Sig.	High income	Low income	Sig.
**Sector**	**Component**	**2007**			**2016**			**2007**			**2016**		
**All 4 sectors combined**	$PM_{2.5}$	2.11 (26.22%)	2.29 (26.86%)	*	1.65 (27.26%)	1.80 (27.92%)	*	0.36 (4.47%)	0.39 (4.62%)	*	0.28 (4.59%)	0.31 (4.75%)	*
	NO_3^-	0.42 (53.44%)	0.46 (53.16%)	*	0.32 (45.33%)	0.35 (45.32%)	*	0.06 (7.63%)	0.06 (7.60%)		0.04 (6.23%)	0.05 (6.25%)	*
	SO_4^{2-}	0.20 (13.83%)	0.21 (13.71%)	*	0.18 (20.68%)	0.19 (20.38%)	*	0.04 (2.95%)	0.05 (2.94%)	*	0.03 (3.84%)	0.04 (3.81%)	*
	NH_4^+	0.22 (35.64%)	0.23 (35.97%)	*	0.13 (36.67%)	0.14 (37.08%)	*	0.03 (5.44%)	0.04 (5.51%)	*	0.02 (5.43%)	0.02 (5.51%)	
	OM	0.94 (22.14%)	1.04 (23.26%)	*	0.72 (24.36%)	0.82 (25.48%)	*	0.19 (4.44%)	0.21 (4.66%)	*	0.14 (4.88%)	0.16 (5.10%)	*
	BC	0.13 (24.24%)	0.15 (25.30%)	*	0.10 (30.45%)	0.12 (31.42%)	*	0.03 (4.85%)	0.03 (5.06%)		0.02 (6.09%)	0.02 (6.29%)	
**Agriculture**	$PM_{2.5}$	1.04 (12.88%)	1.08 (12.71%)	*	0.74 (12.26%)	0.78 (12.11%)	*	0.15 (1.84%)	0.15 (1.82%)		0.10 (1.65%)	0.10 (1.63%)	
	NO_3^-	0.33 (40.79%)	0.35 (40.35%)	*	0.24 (33.66%)	0.26 (33.27%)	*	0.04 (5.23%)	0.04 (5.16%)		0.03 (3.94%)	0.03 (3.88%)	
	SO_4^{2-}	0.16 (11.33%)	0.17 (11.28%)	*	0.13 (15.28%)	0.14 (14.88%)	*	0.03 (2.46%)	0.04 (2.46%)	*	0.03 (3.05%)	0.03 (3.02%)	
	NH_4^+	0.17 (28.26%)	0.18 (28.37%)	*	0.10 (27.50%)	0.10 (27.50%)		0.02 (4.02%)	0.03 (4.04%)	*	0.01 (3.75%)	0.01 (3.74%)	
	OM	0.03 (0.80%)	0.03 (0.75%)		0.02 (0.77%)	0.02 (0.77%)		0.01 (0.15%)	0.01 (0.14%)		0 (0.15%)	0.01 (0.15%)	*
	BC	0 (0.12%)	0 (0.11%)		0 (0.19%)	0 (0.18%)		n/a	n/a	-	0 (0.03%)	0 (0.03%)	
**Transportation**	$PM_{2.5}$	0.49 (6.19%)	0.55 (6.54%)	*	0.34 (5.71%)	0.38 (6.10%)	*	0.10 (1.24%)	0.11 (1.31%)	*	0.07 (1.14%)	0.08 (1.22%)	*
	NO_3^-	0.09 (12.65%)	0.11 (12.71%)	*	0.08 (11.66%)	0.09 (11.97%)	*	0.02 (2.53%)	0.02 (2.54%)		0.02 (2.33%)	0.02 (2.39%)	
	SO_4^{2-}	0.02 (1.29%)	0.02 (1.29%)		0.01 (1.07%)	0.01 (0.99%)		0 (0.26%)	0 (0.20%)		0 (0.21%)	0 (0.20%)	
	NH_4^+	0.03 (5.33%)	0.04 (5.57%)	*	0.02 (6.16%)	0.02 (6.61%)		0.01 (1.07%)	0.01 (1.11%)		0 (1.23%)	0 (1.32%)	
	OM	0.30 (7.29%)	0.32 (7.63%)	*	0.16 (5.81%)	0.17 (6.17%)	*	0.06 (1.46%)	0.06 (1.53%)		0.03 (1.16%)	0.03 (1.23%)	
	BC	0.10 (17.80%)	0.12 (18.70%)	*	0.06 (18.48%)	0.08 (19.41%)	*	0.02 (3.56%)	0.02 (3.74%)		0.01 (3.70%)	0.02 (3.88%)	*
**RWC**	$PM_{2.5}$	0.51 (6.25%)	0.58 (6.73%)	*	0.51 (8.24%)	0.59 (8.74%)	*	0.10 (1.25%)	0.12 (1.35%)	*	0.10 (1.65%)	0.12 (1.75%)	*
	NO_3^-	0 (0.37%)	0 (0.39%)		0 (0.35%)	0 (0.33%)		0 (0.07%)	0 (0.08%)		0 (0.07%)	0 (0.07%)	
	SO_4^{2-}	n/a	n/a	-	0 (0.50%)	0 (0.54%)		n/a	n/a	-	0 (0.10%)	0 (0.11%)	
	NH_4^+	0 (0.18%)	0 (0.20%)		0 (0.39%)	0 (0.39%)		0 (0.04%)	n/a	-	0 (0.08%)	0 (0.08%)	
	OM	0.61 (14.04%)	0.69 (14.87%)	*	0.54 (17.76%)	0.62 (18.53%)	*	0.12 (2.81%)	0.14 (2.97%)	*	0.11 (3.55%)	0.12 (3.71%)	*
	BC	0.03 (6.30%)	0.04 (6.48%)	*	0.04 (11.74%)	0.04 (11.81%)		0.01 (1.26%)	0.01 (1.30%)		0.01 (2.35%)	0.01 (2.36%)	
**Mining**	$PM_{2.5}$	0.07 (0.91%)	0.07 (0.88%)		0.06 (1.05%)	0.06 (0.97%)		0.01 (0.18%)	0.01 (0.18%)		0.01 (0.21%)	0.01 (0.19%)	
	NO_3^-	0 (0.37%)	0 (0.31%)		0 (0.35%)	0 (0.25%)		0 (0.07%)	0 (0.07%)		0 (0.07%)	0 (0.05%)	
	SO_4^{2-}	0.06 (3.79%)	0.06 (3.72%)		0.05 (5.96%)	0.06 (5.95%)	*	0.01 (0.76%)	0.01 (0.74%)		0.01 (1.19%)	0.01 (1.19%)	
	NH_4^+	0.01 (1.87%)	0.01 (1.83%)		0.01 (2.61%)	0.01 (2.58%)		0 (0.37%)	0 (0.37%)		0 (0.52%)	0 (0.52%)	
	OM	n/a	n/a	-	n/a	n/a	-	n/a	n/a	-	n/a	n/a	-
	BC	n/a	n/a	-	n/a	n/a	-	n/a	n/a	-	n/a	n/a	-

Note: BC- Black carbon; NH_4^+- Ammonium; NO_3^-- Nitrate; OM- Organic matter; RWC – Residential wood combustion; SO_4^{2-}—Sulfate. "Sig" is significance based on $p < 0.05$.

Table 4S. Estimated reduction in exposure of total mass $PM_{2.5}$ and its five major components for non-seniors (under 60 years of age) versus seniors (60+ years of age) groups by four selected emission sectors and combined for 100% and 20% scenarios between 2007 and 2016.

0		100% reduction (μg/m^3)						20% reduction (μg/m^3)					
		Non-seniors	Seniors	Sig.	Non-seniors	Seniors	Sig.	Non-seniors	Seniors	Sig.	Non-seniors	Seniors	Sig.
**Sector**	**Component**	**2007**			**2016**			**2007**			**2016**		
**All 4 sectors combined**	$PM_{2.5}$	2.10 (26.12%)	2.08 (25.84%)	*	1.65 (27.15%)	1.62 (26.85%)	*	0.36 (4.46%)	0.36 (4.43%)		0.28 (4.57%)	0.28 (4.57%)	
	NO_3^-	0.42 (53.25%)	0.41 (52.67%)	*	0.33 (45.28%)	0.31 (44.43%)	*	0.06 (7.61%)	0.06 (7.59%)		0.04 (6.22%)	0.04 (6.19%)	
	SO_4^{2-}	0.20 (13.80%)	0.20 (13.51%)		0.18 (20.66%)	0.17 (20.00%)	*	0.04 (2.95%)	0.04 (2.89%)		0.03 (3.82%)	0.03 (3.73%)	
	NH_4^+	0.22 (35.55%)	0.21 (34.97%)	*	0.13 (36.68%)	0.12 (35.76%)	*	0.03 (5.42%)	0.03 (5.37%)		0.02 (5.42%)	0.02 (5.35%)	
	OM	0.93 (22.02%)	0.94 (22.20%)	*	0.71 (24.08%)	0.73 (24.71%)	*	0.19 (4.41%)	0.19 (4.45%)		0.14 (4.82%)	0.15 (4.95%)	
	BC	0.13 (24.32%)	0.13 (23.52%)		0.10 (30.38%)	0.10 (29.93%)		0.03 (5.42%)	0.03 (4.45%)		0.02 (6.08%)	0.02 (5.99%)	
**Agriculture**	$PM_{2.5}$	1.04 (12.84%)	1.00 (12.46%)	*	0.74 (12.27%)	0.71 (11.74%)	*	0.15 (1.83%)	0.14 (1.79%)	*	0.10 (1.65%)	0.10 (1.60%)	
	NO_3^-	0.33 (40.65%)	0.32 (39.97%)	*	0.25 (33.65%)	0.23 (32.80%)	*	0.04 (5.22%)	0.04 (5.19%)		0.03 (3.93%)	0.03 (3.91%)	
	SO_4^{2-}	0.16 (11.59%)	0.16 (10.97%)		0.13 (15.29%)	0.12 (14.52%)	*	0.03 (2.45%)	0.03 (2.38%)		0.03 (3.04%)	0.03 (2.93%)	
	NH_4^+	0.17 (28.19%)	0.17 (27.56%)		0.10 (27.53%)	0.09 (26.59%)	*	0.02 (4.01%)	0.02 (3.95%)		0.01 (3.74%)	0.01 (3.67%)	
	OM	0.03 (0.80%)	0.03 (0.76%)		0.02 (0.78%)	0.02 (0.72%)		0.01 (0.15%)	0.01 (0.14%)		0 (0.15%)	0 (0.14%)	
	BC	0 (0.12%)	0 (0.12%)		0 (0.19%)	0 (0.18%)		0 (0.01%)	0 (0.01%)		0 (0.03%)	0 (0.03%)	
**Transportation**	$PM_{2.5}$	0.50 (6.20%)	0.48 (6.07%)	*	0.34 (5.74%)	0.33 (5.57%)	*	0.10 (1.24%)	0.10 (1.21%)		0.07 (1.15%)	0.07 (1.11%)	
	NO_3^-	0.09 (12.60%)	0.09 (12.71%)		0.08 (11.63%)	0.08 (11.67%)		0.02 (2.52%)	0.02 (2.54%)		0.02 (2.33%)	0.02 (2.33%)	
	SO_4^{2-}	0.02 (1.29%)	0.02 (1.30%)		0.01 (1.05%)	0.01 (1.08%)		0 (0.26%)	0 (0.26%)		0 (0.21%)	0 (0.22%)	
	NH_4^+	0.03 (5.32%)	0.03 (5.32%)		0.02 (6.17%)	0.02 (6.09%)		0.01 (1.06%)	0.01 (1.06%)		0 (1.23%)	0 (1.22%)	
	OM	0.30 (7.33%)	0.29 (7.08%)	*	0.16 (5.86%)	0.15 (5.65%)	*	0.06 (1.47%)	0.06 (1.42%)		0.03 (1.17%)	0.03 (1.13%)	
	BC	0.10 (17.97%)	0.09 (16.92%)	*	0.06 (18.71%)	0.06 (17.45%)		0.02 (3.59%)	0.02 (3.38%)		0.01 (3.74%)	0.01 (3.49%)	
**RWC**	$PM_{2.5}$	0.50 (6.17%)	0.52 (6.40%)	*	0.51 (8.10%)	0.53 (8.48%)	*	0.10 (1.23%)	0.10 (1.28%)	*	0.10 (1.62%)	0.11 (1.70%)	*
	NO_3^-	0 (0.37%)	0 (0.40%)		0 (0.33%)	0 (0.38%)		0 (0.07%)	0 (0.08%)		0 (0.07%)	0 (0.08%)	
	SO_4^{2-}	n/a	n/a	-	0 (0.50%)	0 (0.52%)		n/a	n/a	-	0 (0.10%)	0 (0.10%)	
	NH_4^+	0 (0.18%)	0 (0.19%)		0 (0.38%)	0 (0.42%)		0 (0.04%)	0 (0.04%)		0 (0.08%)	0 (0.08%)	
	OM	0.60 (13.88%)	0.63 (14.34%)	*	0.53 (17.42%)	0.56 (18.31%)	*	0.12 (2.78%)	0.13 (2.87%)	*	0.11 (3.48%)	0.11 (3.66%)	*
	BC	0.03 (6.21%)	0.03 (6.47%)		0.04 (11.45%)	0.04 (12.27%)		0.01 (1.24%)	0.01 (1.29%)		0.01 (2.29%)	0.01 (2.45%)	
**Mining**	$PM_{2.5}$	0.07 (0.91%)	0.07 (0.92%)		0.06 (1.04%)	0.06 (1.06%)		0.01 (0.18%)	0.01 (0.18%)		0.01 (0.21%)	0.01 (0.21%)	
	NO_3^-	0 (0.37%)	0 (0.41%)	*	0 (0.33%)	0 (0.41%)	*	0 (0.07%)	0 (0.08%)		0 (0.07%)	0 (0.08%)	
	SO_4^{2-}	0.06 (3.79%)	0.06 (3.83%)		0.05 (5.92%)	0.05 (6.04%)		0.01 (0.76%)	0.01 (0.77%)	*	0.01 (1.18%)	0.01 (1.21%)	
	NH_4^+	0.01 (1.87%)	0.01 (1.89%)		0.01 (2.59%)	0.01 (2.66%)		0 (0.37%)	0 (0.38%)	*	0 (0.52%)	0 (0.53%)	
	OM	n/a	n/a	-	0 (0.04%)	0 (0.03%)		n/a	n/a	-	n/a	n/a	-
	BC	n/a	n/a	-	n/a	n/a	-	n/a	n/a	-	n/a	n/a	-

Note: BC- Black carbon; NH_4^+- Ammonium; NO_3^-- Nitrate; OM- Organic matter; RWC – Residential wood combustion; SO_4^{2-}–Sulfate. “Sig” is significance based on $p < 0.05$.

Table 5S. Estimated reduction in exposure of total mass $PM_{2.5}$ and its five major components for immigrants versus non-immigrants by birth groups by four selected emission sectors and combined for 100% and 20% scenarios between 2007 and 2016.

		100% reduction (μg/m^3)						20% reduction (μg/m^3)					
		Non-immigrant	Immigrant	Sig.	Non-immigrant	Immigrant	Sig.	Non-immigrant	Immigrant	Sig.	Non-immigrant	Immigrant	Sig.
**Sector**	**Component**	**2007**			**2016**			**2007**			**2016**		
**All 4 sectors combined**	$PM_{2.5}$	2.06 (26.11%)	2.32 (26.41%)	*	1.63 (27.13%)	1.72 (27.45%)	*	0.35 (4.46%)	0.40 (4.50%)	*	0.28 (4.59%)	0.29 (4.58%)	*
	NO_3^-	0.40 (53.35%)	0.51 (52.98%)	*	0.31 (44.97%)	0.37 (45.96%)	*	0.06 (7.63%)	0.07 (7.56%)	*	0.04 (6.21%)	0.05 (6.25%)	*
	SO_4^{2-}	0.21 (14.21%)	0.20 (12.07%)	*	0.17 (20.75%)	0.18 (19.83%)	*	0.04 (3.02%)	0.04 (2.64%)		0.03 (3.85%)	0.03 (3.68%)	
	NH_4^+	0.21 (35.43%)	0.24 (35.99%)	*	0.12 (36.20%)	0.14 (37.81%)	*	0.03 (5.41%)	0.04 (5.49%)	*	0.02 (5.38%)	0.02 (5.52%)	
	OM	0.94 (22.30%)	0.95 (21.59%)	*	0.72 (24.67%)	0.71 (23.34%)	*	0.19 (4.47%)	0.19 (4.32%)		0.14 (4.94%)	0.14 (4.68%)	
	BC	0.12 (23.89%)	0.17 (25.82%)	*	0.09 (30.38%)	0.12 (30.63%)	*	0.02 (4.78%)	0.03 (5.16%)	*	0.02 (6.08%)	0.02 (6.13%)	
**Agriculture**	$PM_{2.5}$	1.00 (12.72%)	1.16 (13.18%)	*	0.72 (12.04%)	0.79 (12.74%)	*	0.14 (1.82%)	0.17 (1.88%)	*	0.10 (1.63%)	0.11 (1.69%)	*
	NO_3^-	0.31 (40.48%)	0.40 (41.21%)	*	0.23 (33.32%)	0.28 (34.25%)	*	0.04 (5.20%)	0.05 (5.29%)	*	0.03 (3.93%)	0.03 (3.93%)	
	SO_4^{2-}	0.16 (11.64%)	0.15 (9.85%)	*	0.13 (15.38%)	0.13 (14.48%)	*	0.04 (2.51%)	0.03 (2.20%)		0.03 (3.06%)	0.03 (2.93%)	
	NH_4^+	0.17 (28.02%)	0.19 (28.73%)	*	0.09 (27.13%)	0.10 (28.32%)	*	0.02 (3.99%)	0.03 (4.08%)	*	0.01 (3.72%)	0.03 (3.77%)	*
	OM	0.03 (0.82%)	0.03 (0.71%)		0.02 (0.76%)	0.02 (0.80%)		0.01 (0.15%)	0.01 (0.13%)		0 (0.15%)	0 (0.16%)	
	BC	0 (0.12%)	0 (0.11%)		0 (0.19%)	0 (0.17%)		0 (0.01%)	n/a		0 (0.03%)	0 (0.02%)	
**Transportation**	$PM_{2.5}$	0.47 (6.05%)	0.59 (6.78%)	*	0.32 (5.52%)	0.39 (6.44%)	*	0.09 (1.21%)	0.12 (1.36%)	*	0.06 (1.10%)	0.08 (1.29%)	*
	NO_3^-	0.09 (12.90%)	0.11 (11.60%)	*	0.08 (11.67%)	0.09 (11.61%)	*	0.02 (2.58%)	0.02 (2.32%)		0.02 (2.33%)	0.02 (2.32%)	
	SO_4^{2-}	0.02 (1.33%)	0.02 (1.11%)		0.01 (1.12%)	0.01 (0.84%)		0 (0.27%)	0 (0.22%)		0 (0.22%)	0 (0.17%)	
	NH_4^+	0.03 (5.30%)	0.04 (5.46%)	*	0.02 (5.99%)	0.02 (6.80%)		0.01 (1.06%)	0.01 (1.09%)		0 (1.20%)	0 (1.36%)	
	OM	0.29 (7.09%)	0.34 (8.12%)	*	0.15 (5.59%)	0.18 (6.63%)	*	0.06 (1.42%)	0.07 (1.62%)	*	0.03 (1.12%)	0.04 (1.33%)	*
	BC	0.09 (17.21%)	0.14 (20.41%)	*	0.06 (17.79%)	0.08 (20.91%)	*	0.02 (3.44%)	0.03 (4.08%)	*	0.01 (3.56%)	0.02 (4.18%)	*
**RWC**	$PM_{2.5}$	0.51 (6.41%)	0.50 (5.65%)	*	0.52 (8.50%)	0.49 (7.39%)		0.10 (1.28%)	0.10 (1.13%)		0.10 (1.70%)	0.10 (1.48%)	
	NO_3^-	0 (0.41%)	0 (0.25%)		0 (0.39%)	0 (0.18%)		0 (0.08%)	0 (0.05%)		0 (0.08%)	0 (0.04%)	
	SO_4^{2-}	n/a	n/a	-	0 (0.50%)	0 (0.53%)		n/a	n/a	-	0 (0.10%)	0 (0.11%)	
	NH_4^+	0 (0.19%)	0 (0.15%)		0 (0.42%)	0 (0.31%)		0 (0.04%)	0 (0.03%)		0 (0.08%)	0 (0.06%)	
	OM	0.62 (14.37%)	0.58 (12.76%)	*	0.55 (18.29%)	0.51 (15.91%)	*	0.12 (2.87%)	0.12 (2.55%)		0.11 (3.66%)	0.10 (3.18%)	*
	BC	0.03 (6.54%)	0.03 (5.30%)		0.04 (12.35%)	0.04 (9.53%)		0.01 (1.31%)	0.01 (1.06%)		0.01 (2.47%)	0.01 (1.91%)	
**Mining**	$PM_{2.5}$	0.07 (0.93%)	0.07 (0.80%)	*	0.06 (1.07%)	0.06 (0.88%)	*	0.01 (0.19%)	0.01 (0.16%)		0.01 (0.21%)	0.01 (0.18%)	
	NO_3^-	0 (0.44%)	0 (0.08%)	*	0 (0.41%)	0 (0.08%)	*	0 (0.09%)	0 (0.02%)		0 (0.08%)	0 (0.02%)	
	SO_4^{2-}	0.06 (3.90%)	0.06 (3.33%)	*	0.05 (6.00%)	0.06 (5.65%)	*	0.01 (0.78%)	0.01 (0.67%)		0.01 (1.20%)	0.01 (1.13%)	
	NH_4^+	0.01 (1.92%)	0.01 (1.65%)		0.01 (2.66%)	0.01 (2.39%)		0 (0.38%)	0 (0.33%)		0 (0.53%)	0 (0.48%)	
	OM	0 (0.10%)	n/a		0 (0.03%)	0 (0.01%)		n/a	n/a	-	n/a	n/a	-
	BC	0 (0.02%)	n/a		0 (0.04%)	0 (0.02%)		n/a	n/a	-	n/a	n/a	-

Note: BC- Black carbon; NH_4^+- Ammonium; NO_3^-- Nitrate; OM- Organic matter; RWC – Residential wood combustion; SO_4^{2-}—Sulfate. "Sig" is significance based on $p < 0.05$.

Table 6S. Estimated reduction in exposure of total mass $PM_{2.5}$ and its five major components for racialized versus non-racialized groups by four selected emission sectors and combined for 100% and 20% scenarios between 2007 and 2016.

		100% reduction (μg/m^3)						20% reduction (μg/m^3)					
		Low minority rate	High minority rate	Sig.	Low minority rate	High minority rate	Sig.	Low minority rate	High minority rate	Sig.	Low minority rate	High minority rate	Sig.
**Sector**	**Component**	**2007**			**2016**			**2007**			**2016**		
**All 4 sectors combined**	$PM_{2.5}$	2.05 (25.93%)	2.37 (26.80%)	*	1.61 (26.85%)	1.75 (27.83%)	*	0.35 (4.43%)	0.40 (4.57%)	*	0.27 (4.55%)	0.29 (4.63%)	*
	NO_3^-	0.40 (53.04%)	0.53 (53.73%)	*	0.31 (44.57%)	0.38 (46.84%)	*	0.06 (7.60%)	0.08 (7.64%)	*	0.04 (6.17%)	0.05 (6.35%)	*
	SO_4^{2-}	0.21 (14.08%)	0.19 (12.04%)	*	0.17 (20.63%)	0.18 (20.02%)	*	0.04 (2.99%)	0.04 (2.65%)		0.03 (3.83%)	0.03 (3.70%)	
	NH_4^+	0.21 (35.19%)	0.24 (36.81%)	*	0.12 (35.82%)	0.14 (38.68%)	*	0.03 (5.38%)	0.04 (5.60%)	*	0.02 (5.34%)	0.02 (5.63%)	
	OM	0.93 (22.10%)	0.97 (21.90%)	*	0.72 (24.45%)	0.70 (23.41%)	*	0.19 (4.43%)	0.19 (4.39%)		0.14 (4.89%)	0.14 (4.69%)	
	BC	0.12 (23.79%)	0.18 (26.24%)	*	0.09 (30.03%)	0.13 (31.14%)	*	0.02 (4.76%)	0.04 (5.25%)	*	0.02 (6.01%)	0.03 (6.23%)	*
**Agriculture**	$PM_{2.5}$	1.00 (12.66%)	1.18 (13.26%)	*	0.71 (11.89%)	0.81 (13.03%)	*	0.14 (1.81%)	0.17 (190%)	*	0.10 (1.61%)	0.11 (1.72%)	*
	NO_3^-	0.31 (40.27%)	0.41 (41.90%)	*	0.23 (33.03%)	0.29 (34.94%)	*	0.04 (5.18%)	0.05 (5.36%)	*	0.03 (3.91%)	0.03 (3.99%)	
	SO_4^{2-}	0.16 (11.48%)	0.15 (9.99%)	*	0.13 (15.20%)	0.13 (14.74%)		0.03 (2.47%)	0.03 (2.24%)		0.03 (3.03%)	0.03 (2.97%)	
	NH_4^+	0.17 (27.82%)	0.19 (29.47%)	*	0.09 (26.82%)	0.11 (29.03%)	*	0.02 (3.97%)	0.03 (4.17%)	*	0.01 (3.69%)	0.01 (3.85%)	
	OM	0.03 (0.81%)	0.03 (0.70%)		0.02 (0.76%)	0.02 (0.80%)		0.01 (0.15%)	0 (0.12%)	*	0 (0.15%)	0 (0.16%)	
	BC	0 (0.12%)	0 (0.10%)		0 (0.19%)	0 (0.16%)		0 (0.01%)	n/a	*	0 (0.03%)	0 (0.02%)	
**Transportation**	$PM_{2.5}$	0.47 (6.01%)	0.62 (7.03%)	*	0.32 (5.43%)	0.40 (6.66%)	*	0.09 (1.20%)	0.12 (1.41%)	*	0.06 (1.09%)	0.08 (1.33%)	*
	NO_3^-	0.09 (12.81%)	0.11 (11.63%)	*	0.08 (11.59%)	0.09 (11.80%)	*	0.02 (2.56%)	0.02 (2.33%)		0.02 (2.32%)	0.02 (2.36%)	
	SO_4^{2-}	0.02 (1.32%)	0.02 (1.14%)		0.01 (1.12%)	0.01 (0.83%)		0 (0.26%)	0 (0.23%)		0 (0.22%)	0 (0.17%)	
	NH_4^+	0.03 (5.26%)	0.04 (5.59%)	*	0.02 (5.91%)	0.02 (7.01%)		0.01 (1.05%)	0.01 (1.12%)		0 (1.18%)	0 (1.40%)	
	OM	0.28 (7.06%)	0.36 (8.47%)	*	0.15 (5.51%)	0.18 (6.89%)	*	0.06 (1.41%)	0.07 (1.69%)	*	0.03 (1.10%)	0.04 (1.38%)	*
	BC	0.09 (17.16%)	0.15 (21.03%)	*	0.05 (17.50%)	0.09 (21.68%)	*	0.02 (3.43%)	0.03 (4.21%)	*	0.01 (3.50%)	0.02 (4.34%)	*
**RWC**	$PM_{2.5}$	0.51 (6.32%)	0.50 (5.65%)	*	0.52 (8.43%)	0.48 (7.28%)	*	0.10 (1.26%)	0.10 (1.13%)		0.10 (1.69%)	0.10 (1.46%)	
	NO_3^-	0 (0.40%)	0 (0.25%)		0 (0.39%)	0 (0.17%)		0 (0.08%)	0 (0.05%)		0 (0.08%)	0 (0.03%)	
	SO_4^{2-}	n/a	n/a	-	0 (0.50%)	0 (0.55%)		n/a	n/a	-	0 (0.10%)	0 (0.11%)	
	NH_4^+	0 (0.19%)	0 (0.15%)		0 (0.42%)	0 (0.29%)		0 (0.04%)	0 (0.03%)		0 (0.08%)	0 (0.06%)	
	OM	0.61 (14.21%)	0.58 (12.73%)	*	0.55 (18.15%)	0.50 (15.72%)	*	0.12 (2.84%)	0.12 (2.55%)		0.11 (3.63%)	0.10 (3.14%)	*
	BC	0.03 (6.48%)	0.03 (5.11%)		0.03 (12.30%)	0.04 (9.28%)	*	0.01 (1.30%)	0.01 (1.02%)		0.01 (2.46%)	0.01 (1.86%)	
**Mining**	$PM_{2.5}$	0.07 (0.94%)	0.07 (0.77%)		0.06 (1.09%)	0.06 (0.86%)		0.01 (0.19%)	0.01 (0.15%)		0.01 (0.22%)	0.01 (0.17%)	
	NO_3^-	0 (0.44%)	0 (0.05%)		0 (0.43%)	0 (0.07%)		0 (0.09%)	0 (0.01%)		0 (0.09%)	0 (0.01%)	
	SO_4^{2-}	0.06 (3.92%)	0.06 (3.19%)	*	0.05 (6.06%)	0.06 (5.57%)	*	0.01 (0.78%)	0.01 (0.64%)	*	0.01 (1.21%)	0.01 (1.11%)	*
	NH_4^+	0.01 (1.92%)	0.01 (1.60%)	*	0.01 (2.68%)	0.01 (2.35%)	*	0 (0.38%)	0 (0.32%)	*	0 (0.54%)	0 (0.47%)	*
	OM	0 (0.01%)	n/a	-	0 (0.03%)	0 (0.01%)		n/a	n/a	-	0 (0.01%)	n/a	*
	BC	0 (0.02%)	n/a	-	0 (0.04%)	0 (0.02%)		n/a	n/a	-	0 (0.01%)	n/a	*

Note: BC- Black carbon; NH_4^+- Ammonium; NO_3^-- Nitrate; OM- Organic matter; RWC – Residential wood combustion; SO_4^{2-}—Sulfate. "Sig" is significance based on $p < 0.05$.

Annual household income *(Figure 2; Table 3S)***:** Under the 100% policy reduction scenario, low-income people would have benefitted more from reductions in emissions originated from agriculture, transportation, and RWC sectors between 2007 and 2016. People with high-income experienced smaller proportional reductions across the same sectors and pollutants. There was no statistical difference between high-income and low-income people residing near active mines and being exposed to related pollutants. In agriculture, the largest reductions were for NO_3^- (~40% or 0.35µg/m^3), NH_4^+ (~28% or 0.18µg/m^3), and SO_4^{2-} (~11% or 0.17µg/m^3) for high and low income groups, but the concentrations for those pollutants were statistically different, except for OM and BC. In the transportation sector, the largest reductions were for BC (~18% pr 0.10µg/m^3 for high-income versus ~19% or 0.12µg/m^3 for low-income) and NO_3^- (~13% for both groups or 0.09µg/m^3 (high income) versus 0.11µg/m^3 (low income)), but the concentrations of SO_4^{2-} were not statistically different between the groups. In RWC, largest reductions were for OM (~14% for high-income versus ~15% for low-income), but the concentrations of NO_3^- and NH_4^+ were not statistically different between the groups. On the other hand, under the 20% policy reduction scenario, the concentrations of SO_4^{2-} and NH_4^+ emitted from the agriculture sector were statistically significant between the two groups in 2007. In 2007, $PM_{2.5}$ was significantly different between the groups for transportation and RWC. In 2016, even less significant differences were found between the low-income and high-income groups.

Age *(Figure 3; Table 4S):* Under the 100% policy reduction scenario, people under 60 years of age would have benefitted more from reductions in emissions originated from agriculture, transportation, and RWC sectors between 2007 and 2016. Older adults (60+ years) experienced smaller proportional reductions across the same sectors. In agriculture, the largest reductions were for NO_3^- (~41% for non-seniors versus ~40% for seniors), NH_4^+ (~28%), and SO_4^{2-} (~12% for non-seniors versus ~11% for seniors), but the concentrations of NO_3^- only was statistically different. In the transportation sector, the largest reductions were for BC (~17-18%) and NO_3^- (~13%), but only the concentrations of BC were statistically different. In RWC, the largest reductions were for OM (~14%). On the other hand, under the 20% policy reduction scenario, in 2007, the concentrations of total $PM_{2.5}$ and its chemicals emitted from the agriculture sector were statistically significant between the two groups. In 2007, OM was significantly different between the groups for RWC. In 2016, significant differences were found between the people under 60 years old and those over 60 years old.

Immigration status *(Figure 4; Table 5S):* Under the 100% policy reduction scenario, immigrants would have benefitted more from reductions in emissions originated from agriculture, transportation, and RWC sectors between 2007 and 2016. People who were born in Canada experienced smaller proportional reductions across the same emission sectors. In agriculture, the largest reductions were for NO_3^- (~41% or 0.31$\mu g/m^3$ (non-immigrants) versus 0.40$\mu g/m^3$ (immigrants)), NH_4^+ (~28%), and SO_4^{2-} (~12% or 0.16$\mu g/m^3$ for non-immigrants versus 10% or 0.15$\mu g/m^3$ for immigrants). In the transportation sector, largest reductions were for BC (~21% or 0.14$\mu g/m^3$ for immigrants versus ~17% or 0.09$\mu g/m^3$ for non-immigrants) and NO_3^- (~13% for non-immigrants versus 12% for immigrants). In RWC, the largest reductions were for OM (~7-8%). On the other hand, under the 20% policy reduction scenario in 2007, concentrations of total $PM_{2.5}$ and its components emitted from the agriculture and transportation sectors were statistically different between the two groups. In the same year, a significant difference was found for concentrations of $PM_{2.5}$, NO_3^- and NH_4^+ produced from agriculture between the two groups. In 2007, for transportation, a significant difference was found for concentrations of $PM_{2.5}$, OM, and BC between the two groups. In 2016, even less significant differences were found between the immigrants and non-immigrants.

Race/ethnicity *(Figure 5; Table 6S)*: Under the 100% policy reduction scenario, visible minorities by race/ethnicity would have benefitted more from reductions in emissions originated from agriculture, transportation, and RWC sectors between 2007 and 2016. Caucasians experienced smaller proportional reductions across the same emission sectors. In agriculture, the largest reductions were for NO_3^- (~40% or 0.31$\mu g/m^3$ for Caucasians versus ~42% or 0.41$\mu g/m^3$ for visible minorities), NH_4^+ (~28% or 0.17$\mu g/m^3$ for Caucasians versus ~29% or 0.19$\mu g/m^3$ for visible minorities), and SO_4^{2-} (~11% or 0.16$\mu g/m^3$ for Caucasians versus ~10% or 0.15$\mu g/m^3$ for visible minorities) in 2007. In the same year, in the transportation sector, the largest reductions were for BC (~17% for Caucasians versus ~21% for visible minorities) and NO_3^- (~13-12%). In 2007, in RWC, the largest reductions were for OM (~14% for Caucasians versus ~13% for visible minorities) and the absolute concentrations were statistically different (0.61 versus 0.58). A similar pattern existed in 2016 between the two subgroups of people. On the other hand, under the 20% policy reduction scenario, concentrations of total $PM_{2.5}$ and its components emitted from the agriculture and transportation sectors were statistically significant between the two groups in 2007. For agriculture, a significant difference was found for concentrations of $PM_{2.5}$, NO_3^-, NH_4^+, OM

and BC between the two groups in 2007. For transportation, a significant difference was found for concentrations of $PM_{2.5}$, OM, and BC between the two groups in 2007. In 2016, even less significant differences were found between the Caucasians and visible minorities.

Education *(Table 7S):* Under the 100% policy reduction scenario, educated people with a university degree or college diploma would have benefitted more from reductions in emissions originated from agriculture, transportation, and RWC sectors between 2007 and 2016. Less educated people would have experienced smaller proportional reductions across the same pollutants. In agriculture, the largest reductions were for NO_3^- (~41% or 0.36μg/m^3 for highly educated people versus ~40% or 0.32μg/m^3 for less educated), NH_4^+ (~29% for highly educated versus ~28% for less educated), and SO_4^{2-} (~11% for both groups). In the transportation sector, the largest reductions were for BC (~19% for highly educated versus ~17% for less educated people) and NO_3^- (~13% for both groups). For RWC sector, the largest reductions were for OM (~14% for both groups). On the other hand, under the 20% policy reduction scenario, the concentrations of total $PM_{2.5}$ and its chemicals emitted from the agricultural, transportation and RWC sectors were statistically significant between the two groups in 2007. In agriculture sector, significant differences were found for the concentrations of $PM_{2.5}$, NO_3^- and NH_4^+ between the two groups in 2007. In transportation sector, significant difference was found for the concentrations of $PM_{2.5}$ between the two groups in 2007. In RWC sector, significant differences were found for the concentrations of $PM_{2.5}$, NH_4^+, and OM between the two groups in 2007. In 2016, even less significant differences were found between the well educated and less educated groups of people.

Employment *(Table 8S):* Under the 100% policy reduction scenario, employed people would have benefitted more from reductions in emissions originated from agriculture, transportation, and RWC sectors between 2007 and 2016. Unemployed people would have experienced smaller proportional reductions across the same pollutants. In agriculture, the largest reductions were for NO_3^- (~41% for employed versus ~39% for unemployed), NH_4^+ (~29% for employed versus ~26% for unemployed), and SO_4^{2-} (~13%). In the transportation sector, the largest reductions were for BC (~18% for employed versus ~17% for unemployed) and NO_3^- (~13% for both groups). In RWC sector, the largest reductions were for OM (~14% for both groups), yet absolute concentrations were not significantly different between the groups. On the other hand, under the 20% policy reduction scenario, the concentrations of total $PM_{2.5}$ and its chemicals emitted from the agriculture

and transportation sectors were statistically significant between the two groups in 2007. In agriculture sector, significant differences were found for the concentrations of $PM_{2.5}$, SO_4^{2-} and OM between the two groups in 2007. In transportation sector, significant difference was found for the concentrations of $PM_{2.5}$ between the two groups in 2007. In 2016, even less significant differences were found between the employed and unemployed groups of people.

Marital status *(Table 9S):* Under the 100% policy reduction scenario, singles would have benefitted more from reductions in emissions originated from agriculture, transportation, and RWC sectors between 2007 and 2016. People categorized as singles (i.e., never married, widowed, divorced or separated) would have experienced smaller proportional reductions across the same pollutants. In the agriculture sector, the largest reductions were for NO_3^- (~41% for singles versus ~40% for married/common-law), NH_4^+ (~28% for both groups), and SO_4^{2-} (~12% for singles versus ~11% for married/common-law), although the concentrations of NO_3^- and NH_4^+ were not statistically significant. In the transportation sector, the largest reductions were for BC (~18% for both groups) and NO_3^- (~13% for singles versus ~12% for married/common-law), but the concentration of BC was not significant between the groups. In the RWC sector, the largest reductions were for OM (~15% for singles versus ~13% for married/common-law). On the other hand, under the 20% policy reduction scenario, the concentrations of total $PM_{2.5}$ and its chemicals emitted from the agriculture, transportation, and RWC sectors were statistically significant between the two groups in 2007. In the agriculture sector, significant differences were found for the concentrations of $PM_{2.5}$ and SO_4^{2-} between the two groups in 2007. In the transportation sector, significant difference was found for the concentrations of BC between the two groups in 2007. For the RWC sector, a significant difference was found for OM between the two groups in 2007. In 2016, even less significant differences were found between the singles and people with the marital status.

Indigenous identity *(Table 10S):* Under the 100% policy reduction scenario, non-Indigenous people would have benefitted more from reductions in emissions originated from the agriculture, transportation, and RWC sectors between 2007 and 2016. Indigenous Peoples would have experienced smaller proportional reductions across the same pollutants. In the agriculture sector, the largest reductions were for NO_3^- (~41% or 0.33μg/m^3 for non-Indigenous versus ~33% or 0.20μg/m^3 for Indigenous), NH_4^+ (~28% or 0.17μg/m^3 for non-Indigenous versus ~22% or

0.10μg/m^3 for Indigenous), and SO_4^{2-} (~11% or 0.16μg/m^3 for non-Indigenous versus ~9% or 0.10μg/m^3 for Indigenous). In the transportation sector, the largest reductions were for BC (~18% or 0.10μg/m^3 for non-Indigenous versus ~11% or 0.07μg/m^3 for Indigenous) and NO_3^- (~13% for non-Indigenous versus ~11% for Indigenous). For the RWC sector, the largest reductions were for OM (~14% or 0.62μg/m^3 for non-Indigenous versus ~7% or 0.29μg/m^3 for Indigenous), yet the absolute concentrations were not significantly different between the groups. On the other hand, under the 20% policy reduction scenario, concentrations of total $PM_{2.5}$ and its chemicals emitted from the agricultural, transportation, and RWC sectors were statistically significant between the two groups in 2007. For the agriculture sector, significant differences were found for the concentrations of $PM_{2.5}$, NO_3^-, SO_4^{2-} and NH_4^+ between the two groups in 2007. For the transportation sector, significant differences were found for concentrations of $PM_{2.5}$, NO_3^-, NH_4^+, and BC between the two groups in 2007. For the RWC sector, significant difference was found for the concentrations of $PM_{2.5}$, OM, and BC between the two groups in 2007. In 2016, even less significant differences were found between the Indigenous Peoples and non-Indigenous people.

Sex at birth *(Table 11S):* No statistical difference was found between males and females exposed to pollutants emitted either from the agriculture, transportation, RWC or mining sectors for both policy reduction scenarios between 2007 and 2016.

Table 7S. Estimated reduction in exposure of total mass $PM_{2.5}$ and its five major components for groups of people with low (i.e., partially or fully completed high school) versus high (i.e., college diploma or university degree (bachelor or above)) educational attainment by four selected emission sectors and combined for 100% and 20% scenarios between 2007 and 2016.

		100% reduction ($\mu g/m^3$)						20% reduction ($\mu g/m^3$)					
		High Education	Low Education	Sig.	High Education	Low Education	Sig.	High Education	Low Education	Sig.	High Education	Low Education	Sig.
**Sector**	**Component**	**2007**			**2016**			**2007**			**2016**		
**All 4 sectors combined**	$PM_{2.5}$	2.24 (26.77%)	2.07 (25.94%)	*	1.72 (27.59%)	1.62 (26.90%)	*	0.38 (4.58%)	0.35 (4.43%)	*	0.29 (4.66%)	0.27 (4.54%)	*
	NO_3^-	0.46 (53.73%)	0.41 (53.05%)	*	0.35 (45.63%)	0.32 (44.84%)	*	0.06 (7.66%)	0.06 (7.60%)		0.05 (6.28%)	0.04 (6.19%)	*
	SO_4^{2-}	0.21 (13.59%)	0.20 (13.77%)	*	0.18 (20.47%)	0.17 (20.40%)	*	0.04 (2.92%)	0.04 (2.94%)		0.03 (3.80%)	0.03 (3.78%)	
	NH_4^+	0.23 (36.13%)	0.21 (35.31%)	*	0.13 (37.14%)	0.12 (36.18%)	*	0.03 (5.53%)	0.03 (5.40%)		0.02 (5.50%)	0.02 (5.37%)	
	OM	0.99 (22.70%)	0.93 (22.01%)	*	0.75 (24.59%)	0.71 (24.21%)	*	0.20 (4.55%)	0.19 (4.41%)	*	0.15 (4.92%)	0.14 (4.85%)	*
	BC	0.15 (25.57%)	0.13 (23.85%)	*	0.11 (31.26%)	0.09 (29.99%)	*	0.03 (5.12%)	0.03 (4.77%)		0.02 (6.25%)	0.02 (6.00%)	
**Agriculture**	$PM_{2.5}$	1.09 (12.96%)	1.02 (12.70%)	*	0.77 (12.36%)	0.72 (12.02%)	*	0.15 (1.85%)	0.14 (1.82%)	*	0.10 (1.67%)	0.10 (1.63%)	
	NO_3^-	0.36 (40.92%)	0.32 (40.43%)	*	0.26 (33.77%)	0.24 (33.28%)	*	0.04 (5.22%)	0.04 (5.21%)		0.03 (3.94%)	0.03 (3.92%)	
	SO_4^{2-}	0.16 (11.21%)	0.13 (11.24%)	*	0.13 (15.08%)	0.13 (14.98%)		0.04 (2.45%)	0.03 (2.44%)	*	0.03 (3.03%)	0.03 (2.99%)	
	NH_4^+	0.18 (28.58%)	0.17 (27.97%)	*	0.10 (27.77%)	0.09 (27.08%)	*	0.03 (4.07%)	0.02 (3.99%)	*	0.01 (3.78%)	0.01 (3.70%)	
	OM	0.03 (0.76%)	0.03 (0.79%)		0.02 (0.78%)	0.02 (0.75%)	*	0.01 (0.14%)	0.01 (0.15%)		0 (0.15%)	0 (0.15%)	
	BC	0 (0.11%)	0 (0.12%)		0 (0.18%)	0 (0.19%)		0 (0.01%)	0 (0.11%)		0 (0.02%)	0 (0.03%)	
**Transportation**	$PM_{2.5}$	0.55 (6.58%)	0.48 (6.09%)	*	0.37 (6.06%)	0.33 (5.60%)	*	0.11 (1.32%)	0.10 (1.22%)	*	0.07 (1.21%)	0.07 (1.12%)	
	NO_3^-	0.10 (12.70%)	0.09 (12.63%)	*	0.09 (11.80%)	0.08 (11.60%)	*	0.02 (2.54%)	0.02(2.53%)		0.02 (2.36%)	0.02 (2.32%)	
	SO_4^{2-}	0.02 (1.25%)	0.02 (1.30%)		0.01 (0.99%)	0.01 (1.07%)		0 (0.25%)	0 (0.26%)		0 (0.20%)	0 (0.21%)	
	NH_4^+	0.04 (5.57%)	0.03 (5.28%)	*	0.02 (6.45%)	0.02 (6.07%)		0.01 (1.11%)	0.01 (1.06%)		0 (1.29%)	0 (1.21%)	
	OM	0.32 (7.71%)	0.29 (7.19%)	*	0.17 (6.22%)	0.16 (5.70%)	*	0.06 (1.54%)	0.06 (1.44%)		0.03 (1.24%)	0.03 (1.14%)	
	BC	0.12 (19.28%)	0.09 (17.40%)	*	0.07 (19.87%)	0.04 (17.93%)	*	0.02 (3.86%)	0.02 (3.48%)		0.01 (3.97%)	0.01 (3.59%)	
**RWC**	$PM_{2.5}$	0.54 (6.38%)	0.50 (6.23%)	*	0.53 (8.19%)	0.51 (8.22%)	*	0.11 (1.28%)	0.10 (1.25%)	*	0.11 (1.64%)	0.10 (1.64%)	*
	NO_3^-	0 (0.36%)	0 (0.38%)		0 (0.30%)	0 (0.36%)		0 (0.07%)	0 (0.08%)		0 (0.06%)	0 (0.07%)	
	SO_4^{2-}	n/a	n/a	-	0 (0.52%)	0 (0.51%)		n/a	n/a	-	0 (0.10%)	0 (0.10%)	
	NH_4^+	0 (0.18%)	0 (0.18%)		0 (0.38%)	0 (0.40%)		n/a	0 (0.04%)	*	0 (0.08%)	0 (0.08%)	
	OM	0.64 (14.22%)	0.61 (14.02%)	*	0.56 (17.58%)	0.54 (17.73%)	*	0.13 (2.84%)	0.12 (2.80%)	*	0.11 (3.52%)	0.11 (3.55%)	
	BC	0.03 (6.18%)	0.03 (6.32%)		0.04 (11.19%)	0.03 (11.83%)	*	0.01 (1.24%)	0.01 (1.26%)		0.01 (2.24%)	0.01 (2.37%)	
**Mining**	$PM_{2.5}$	0.07 (0.86%)	0.07 (0.92%)		0.06 (0.98%)	0.06 (1.06%)		0.01 (0.17%)	0.01 (0.18%)	*	0.01 (0.20%)	0.01 (0.21%)	*
	NO_3^-	0 (0.25%)	0 (0.41%)		0 (0.24%)	0 (0.39%)		0 (0.05%)	0 (0.08%)		0 (0.05%)	0 (0.08%)	
	SO_4^{2-}	0.06 (3.63%)	0.06 (3.82%)		0.06 (5.86%)	0.05 (5.98%)	*	0.01 (0.73%)	0.01 (0.76%)		0.01 (1.17%)	0.01 (1.20%)	
	NH_4^+	0.01 (1.80%)	0.01 (1.88%)		0.01 (2.54%)	0.01 (2.63%)		0 (0.36%)	0 (0.38%)		0 (0.51%)	0 (0.53%)	
	OM	n/a	n/a	-	n/a	0 (0.03%)	*	n/a	n/a	-	n/a	n/a	-
	BC	n/a	n/a	-	n/a	0 (0.04%)	*	n/a	n/a	-	n/a	n/a	-

Note: BC- Black carbon; NH_4^+- Ammonium; NO_3^-- Nitrate; OM- Organic matter; RWC – Residential wood combustion; SO_4^{2-}—Sulfate. "Sig" is significance based on $p < 0.05$.

Table 8S. Estimated reduction in exposure of total mass $PM_{2.5}$ and its five major components for employed versus unemployed groups by four selected emission sectors and combined for 100% and 20% scenarios between 2007 and 2016.

		100% reduction (μg/m^3)						20% reduction (μg/m^3)					
		Employed	Unemployed	Sig.	Employed	Unemployed	Sig.	Employed	Unemployed	Sig.	Employed	Unemployed	Sig.
**Sector**	**Component**	**2007**			**2016**			**2007**			**2016**		
**All 4 sectors combined**	$PM_{2.5}$	2.13 (26.37%)	2.01 (24.81%)	*	1.66 (27.33%)	1.61 (26.32%)	*	0.36 (4.49%)	0.34 (4.29%)	*	0.28 (4.60%)	0.27 (4.44%)	*
	NO_3^-	0.43 (53.64%)	0.39 (51.58%)	*	0.33 (45.51%)	0.32 (44.71%)	*	0.06 (7.63%)	0.06 (7.59%)		0.04 (6.24%)	0.04 (6.22%)	
	SO_4^{2-}	0.20 (13.95%)	0.20 (13.00%)	*	0.18 (20.71%)	0.17 (20.41%)	*	0.04 (2.98%)	0.04 (2.79%)		0.03 (3.83%)	0.03 (3.77%)	
	NH_4^+	0.22 (35.93%)	0.21 (33.58%)	*	0.13 (36.88%)	0.12 (35.84%)	*	0.03 (5.47%)	0.03 (5.22%)		0.02 (5.45%)	0.02 (5.33%)	
	OM	0.94 (22.15%)	0.91 (21.69%)	*	0.72 (24.30%)	0.70 (23.40%)	*	0.19 (4.44%)	0.18 (4.35%)	*	0.14 (4.87%)	0.14 (4.68%)	
	BC	0.13 (24.54%)	0.13 (23.23%)		0.10 (30.58%)	0.10 (30.01%)		0.03 (4.91%)	0.03 (4.65%)		0.02 (6.12%)	0.02 (6.01%)	
**Agriculture**	$PM_{2.5}$	1.05 (13.03%)	0.96 (11.75%)	*	0.75 (12.34%)	0.73 (11.98%)	*	0.15 (1.86%)	0.14 (1.71%)	*	0.10 (1.66%)	0.10 (1.63%)	
	NO_3^-	0.33 (40.96%)	0.30 (39.23%)	*	0.25 (33.78%)	0.24 (33.49%)	*	0.04 (5.22%)	0.04 (5.26%)		0.03 (3.94%)	0.03 (4.01%)	
	SO_4^{2-}	0.16 (12.51%)	0.15 (10.25%)	*	0.13 (15.36%)	0.13 (15.09%)		0.04 (2.49%)	0.03 (2.24%)	*	0.03 (3.06%)	0.03 (2.98%)	
	NH_4^+	0.17 (28.54%)	0.16 (26.41%)	*	0.10 (27.69%)	0.09 (27.02%)	*	0.02 (4.05%)	0.02 (3.84%)		0.01 (3.76%)	0.01 (3.72%)	
	OM	0.03 (0.83%)	0.03 (0.68%)		0.02 (0.78%)	0.02 (0.74%)		0.01 (0.15%)	0 (0.12%)	*	0 (0.15%)	0 (0.14%)	
	BC	0 (0.12%)	0 (0.10%)		0 (0.19%)	0 (0.18%)		0 (0.01%)	0 (0.01%)		0 (0.03%)	0 (0.02%)	
**Transportation**	$PM_{2.5}$	0.50 (6.27%)	0.47 (5.83%)	*	0.34 (5.79%)	0.33 (5.50%)	*	0.10 (1.25%)	0.09 (1.17%)	*	0.07 (1.16%)	0.07 (1.10%)	
	NO_3^-	0.10 (12.65%)	0.09 (12.52%)	*	0.08 (11.70%)	0.08 (11.34%)		0.02 (2.53%)	0.02 (2.50%)		0.02 (2.34%)	0.02 (2.27%)	
	SO_4^{2-}	0.02 (1.30%)	0.02 (1.19%)		0.01 (1.06%)	0.01 (1.03%)		0 (0.26%)	0 (0.24%)		0 (0.21%)	0 (0.21%)	
	NH_4^+	0.03 (5.37%)	0.03 (5.04%)		0.02 (6.23%)	0.02 (5.81%)		0.01 (1.07%)	0.01 (1.01%)		0 (1.25%)	0 (1.16%)	
	OM	0.30 (7.42%)	0.28 (6.85%)	*	0.16 (5.90%)	0.16 (5.74%)		0.06 (1.48%)	0.06 (1.37%)		0.03 (1.18%)	0.03 (1.15%)	
	BC	0.10 (18.20%)	0.09 (16.69%)	*	0.06 (18.80%)	0.06 (18.48%)		0.02 (3.64%)	0.02 (3.34%)		0.01 (3.76%)	0.01 (3.70%)	
**RWC**	$PM_{2.5}$	0.50 (6.18%)	0.51 (6.29%)	*	0.51 (8.18%)	0.49 (7.79%)	*	0.10 (1.24%)	0.10 (1.26%)		0.10 (1.64%)	0.10 (1.56%)	
	NO_3^-	0 (0.36%)	0 (0.42%)		0 (0.34%)	0 (0.34%)		0 (0.07%)	0 (0.08%)		0 (0.07%)	0 (0.07%)	
	SO_4^{2-}	n/a	n/a	-	0 (0.52%)	0 (0.47%)		n/a	n/a	-	0 (0.10%)	0 (0.09%)	
	NH_4^+	0 (0.18%)	0 (0.18%)		0 (0.39%)	0 (0.38%)		0 (0.04%)	0 (0.04%)		0 (0.08%)	0 (0.08%)	
	OM	0.61 (13.89%)	0.61 (14.15%)		0.54 (17.60%)	0.52 (16.88%)	*	0.12 (2.78%)	0.12 (2.83%)		0.11 (3.52%)	0.10 (3.38%)	*
	BC	0.03 (6.20%)	0.03 (6.42%)		0.04 (11.55%)	0.03 (11.32%)	*	0.01 (1.24%)	0.01 (1.28%)		0.01 (2.31%)	0.01 (2.26%)	
**Mining**	$PM_{2.5}$	0.07 (0.90%)	0.07 (0.95%)		0.06 (1.03%)	0.06 (1.05%)		0.01 (0.18%)	0.01 (0.19%)		0.01 (0.21%)	0.01 (0.21%)	
	NO_3^-	0 (0.33%)	0 (0.60%)		0 (0.32%)	0 (0.46%)		0 (0.07%)	0 (0.12%)		0 (0.06%)	0 (0.09%)	
	SO_4^{2-}	0.06 (3.74%)	0.06 (3.94%)		0.05 (5.89%)	0.05 (5.88%)		0.01 (0.75%)	0.01 (0.79%)		0.01 (1.18%)	0.01 (1.18%)	
	NH_4^+	0.01 (1.84%)	0.01 (1.95%)		0.01 (2.57%)	0.01 (2.63%)		0 (0.37%)	0 (0.39%)		0 (0.51%)	0 (0.53%)	
	OM	n/a	n/a	-	0 (0.02%)	0 (0.04%)		n/a	n/a	-	n/a	n/a	-
	BC	n/a	n/a	-	0 (0.04%)	0 (0.03%)		n/a	n/a	-	n/a	n/a	-

Note: BC- Black carbon; NH_4^+- Ammonium; NO_3^-- Nitrate; OM- Organic matter; RWC – Residential wood combustion; SO_4^{2-}—Sulfate. "Sig" is significance based on $p < 0.05$.

Table 9S. Estimated reduction in exposure of total mass $PM_{2.5}$ and its five major components for in status (married or common-law) versus singles (never married, was widowed, divorced or separated) groups by four selected emission sectors and combined for 100% and 20% scenarios between 2007 and 2016.

		100% reduction (μg/m^3)						20% reduction (μg/m^3)					
		Single	In status	Sig.	Single	In status	Sig.	Single	In status	Sig.	Single	In status	Sig.
**Sector**	**Component**	**2007**			**2016**			**2007**			**2016**		
**All 4 sectors combined**	$PM_{2.5}$	2.17 (26.67%)	2.03 (25.44%)	*	1.69 (27.20%)	1.55 (26.25%)	*	0.37 (4.57%)	0.35 (4.34%)	*	0.29 (4.60%)	0.26 (4.40%)	*
	NO_3^-	0.43 (53.61%)	0.42 (52.66%)	*	0.33 (44.89%)	0.32 (44.79%)	*	0.06 (7.63%)	0.06 (7.59%)		0.04 (6.20%)	0.04 (6.20%)	
	SO_4^{2-}	0.21 (14.33%)	0.19 (13.10%)	*	0.18 (20.49%)	0.17 (19.72%)	*	0.05 (3.06%)	0.04 (2.80%)	*	0.03 (3.81%)	0.03 (3.62%)	
	NH_4^+	0.22 (35.89%)	0.21 (34.96%)	*	0.13 (36.37%)	0.12 (36.10%)	*	0.03 (5.48%)	0.03 (5.35%)		0.02 (5.40%)	0.02 (5.33%)	
	OM	0.99 (22.94%)	0.88 (21.25%)	*	0.75 (24.58%)	0.66 (22.92%)	*	0.20 (4.60%)	0.18 (4.26%)	*	0.15 (4.92%)	0.13 (4.59%)	*
	BC	0.13 (24.45%)	0.13 (23.81%)		0.10 (30.48%)	0.10 (29.33%)		0.03 (4.89%)	0.03 (4.76%)		0.02 (6.10%)	0.02 (5.87%)	
**Agriculture**	$PM_{2.5}$	1.04 (12.77%)	1.02 (12.69%)	*	0.74 (12.04%)	0.72 (12.12%)	*	0.15 (1.82%)	0.14 (1.82%)	*	0.10 (1.63%)	0.10 (1.63%)	*
	NO_3^-	0.33 (40.58%)	0.33 (40.42%)		0.25 (33.21%)	0.24 (33.45%)	*	0.04 (5.17%)	0.04 (5.27%)	*	0.03 (3.90%)	0.03 (3.97%)	*
	SO_4^{2-}	0.17 (11.83%)	0.15 (10.59%)	*	0.13 (15.04%)	0.12 (14.37%)	*	0.04 (2.56%)	0.03 (2.30%)	*	0.03 (3.02%)	0.02 (2.86%)	*
	NH_4^+	0.17 (28.34%)	0.17 (27.77%)		0.10 (27.16%)	0.09 (27.08%)	*	0.02 (4.04%)	0.02 (3.97%)		0.01 (3.71%)	0.01 (3.68%)	
	OM	0.03 (0.77%)	0.03 (0.81%)		0.02 (0.76%)	0.02 (0.77%)		0.01 (0.14%)	0.01 (0.15%)		0 (0.15%)	0 (0.15%)	
	BC	0 (0.12%)	0 (0.12%)		0 (0.18%)	0 (0.18%)		0 (0.01%)	0 (0.01%)		0 (0.03%)	0 (0.03%)	
**Transportation**	$PM_{2.5}$	0.50 (6.23%)	0.49 (6.12%)	*	0.34 (5.73%)	0.33 (5.71%)	*	0.10 (1.25%)	0.10 (1.22%)		0.07 (1.15%)	0.07 (1.14%)	
	NO_3^-	0.10 (12.99%)	0.09 (12.28%)	*	0.08 (11.68%)	0.08 (11.40%)		0.02 (2.60%)	0.02 (2.46%)		0.02 (2.34%)	0.02 (2.28%)	
	SO_4^{2-}	0.02 (1.35%)	0.02 (1.23%)		0.01 (1.05%)	0.01 (0.98%)		0 (0.27%)	0 (0.25%)		0 (0.21%)	0 (0.20%)	
	NH_4^+	0.03 (5.45%)	0.03 (5.20%)		0.02 (6.19%)	0.02 (6.12%)		0.01 (1.09%)	0.01 (1.04%)		0 (1.24%)	0 (1.22%)	
	OM	0.30 (7.20%)	0.29 (7.36%)	*	0.16 (5.82%)	0.16 (5.93%)		0.06 (1.44%)	0.06 (1.47%)		0.03 (1.16%)	0.03 (1.19%)	
	BC	0.10 (17.62%)	0.10 (17.82%)		0.07 (18.43%)	0.06 (18.51%)	*	0.02 (3.52%)	0.02 (3.56%)		0.01 (3.69%)	0.01 (3.70%)	
**RWC**	$PM_{2.5}$	0.56 (6.75%)	0.46 (5.73%)	*	0.54 (8.39%)	0.45 (7.40%)	*	0.11 (1.35%)	0.09 (1.15%)	*	0.11 (1.68%)	0.09 (1.48%)	*
	NO_3^-	0 (0.41%)	0 (0.34%)		0 (0.35%)	0 (0.29%)		0 (0.08%)	0 (0.07%)		0 (0.07%)	0 (0.06%)	
	SO_4^{2-}	n/a	n/a	-	0 (0.51%)	0 (0.52%)		n/a	n/a	-	0 (0.10%)	0 (0.10%)	
	NH_4^+	0 (0.20%)	0 (0.16%)		0 (0.40%)	0 (0.36%)		0 (0.04%)	0 (0.03%)		0 (0.08%)	0 (0.07%)	
	OM	0.66 (14.96%)	0.56 (13.08%)	*	0.56 (17.99%)	0.48 (16.20%)	*	0.13 (2.99%)	0.11 (2.62%)	*	0.11 (3.60%)	0.10 (3.24%)	*
	BC	0.03 (6.71%)	0.03 (5.85%)		0.04 (11.83%)	0.03 (10.60%)	*	0.01 (1.34%)	0.01 (1.17%)		0.01 (2.37%)	0.01 (2.12%)	
**Mining**	$PM_{2.5}$	0.07 (0.91%)	0.07 (0.90%)		0.06 (1.04%)	0.06 (1.02%)		0.01 (0.18%)	0.01 (0.18%)		0.01 (0.21%)	0.01 (0.20%)	
	NO_3^-	0 (0.38%)	0 (0.38%)		0 (0.35%)	0 (0.35%)		0 (0.08%)	0 (0.08%)		0 (0.07%)	0 (0.07%)	
	SO_4^{2-}	0.06 (3.85%)	0.06 (3.73%)	*	0.06 (5.99%)	0.05 (5.81%)	*	0.01 (0.77%)	0.01 (0.75%)		0.01 (1.20%)	0.01 (1.16%)	
	NH_4^+	0.01 (1.89%)	0.01 (1.84%)		0.01 (2.62%)	0.01 (2.54%)		0 (0.38%)	0 (0.37%)		0 (0.52%)	0 (0.51%)	
	OM	0 (0.01%)	0 (0.01%)		0 (0.02%)	0 (0.03%)		n/a	n/a	-	n/a	0 (0.01%)	*
	BC	0 (0.01%)	0 (0.01%)		0 (0.03%)	0 (0.04%)		n/a	n/a	-	0 (0.01%)	0 (0.01%)	

Note: BC- Black carbon; NH_4^+- Ammonium; NO_3^-- Nitrate; OM- Organic matter; RWC – Residential wood combustion; SO_4^{2-}–Sulfate. "Sig" is significance based on $p < 0.05$.

Table 10S. Estimated reduction in exposure of total mass $PM_{2.5}$ and its five major components for Indigenous Peoples (belonging to one or more of the following groups: Métis, Inuit, First Nations) versus non-Indigenous groups by four selected emission sectors and combined for 100% and 20% scenarios between 2007 and 2016.

		100% reduction (μg/m³)						20% reduction (μg/m³)					
		Non-Indigeneous	Indigeneous	Sig.	Non-Indigeneous	Indigeneous	Sig.	Non-Indigeneous	Indigeneous	Sig.	Non-Indigeneous	Indigeneous	Sig.
**Sector**	**Component**	**2007**			**2016**			**2007**			**2016**		
**All 4 sectors combined**	$PM_{2.5}$	2.13 (26.34%)	1.25 (18.88%)	*	1.67 (27.45%)	1.09 (19.61%)	*	0.36 (4.50%)	0.21 (3.27%)	*	0.28 (4.63%)	0.18 (3.31%)	*
	NO_3^-	0.43 (53.53%)	0.27 (42.94%)	*	0.33 (45.43%)	0.23 (38.13%)	*	0.06 (7.64%)	0.04 (6.66%)	*	0.04 (6.23%)	0.03 (5.72%)	*
	SO_4^{2-}	0.21 (13.79%)	0.15 (12.59%)	*	0.18 (20.67%)	0.13 (17.04%)	*	0.04 (2.95%)	0.03 (2.53%)	*	0.03 (3.85%)	0.02 (2.93%)	*
	NH_4^+	0.22 (35.71%)	0.13 (28.46%)	*	0.13 (36.80%)	0.09 (29.78%)	*	0.03 (5.45%)	0.02 (4.53%)	*	0.02 (5.44%)	0.01 (4.58%)	*
	OM	0.95 (22.31%)	0.57 (15.29%)	*	0.73 (24.62%)	0.45 (16.13%)	*	0.19 (4.47%)	0.11 (3.06%)	*	0.15 (4.93%)	0.09 (3.23%)	*
	BC	0.13 (24.39%)	0.07 (18.63%)	*	0.10 (30.65%)	0.06 (22.88%)	*	0.03 (4.88%)	0.01 (3.74%)	*	0.02 (6.13%)	0.01 (4.58%)	*
**Agriculture**	$PM_{2.5}$	1.04 (12.88%)	0.65 (9.79%)	*	0.74 (12.28%)	0.55 (9.46%)	*	0.15 (1.84%)	0.10 (1.48%)	*	0.10 (1.65%)	0.08 (1.35%)	*
	NO_3^-	0.33 (40.82%)	0.20 (32.63%)	*	0.25 (33.69%)	0.18 (28.71%)	*	0.04 (5.23%)	0.03 (4.71%)	*	0.03 (3.93%)	0.02 (3.83%)	*
	SO_4^{2-}	0.16 (11.33%)	0.10 (8.67%)	*	0.13 (15.29%)	0.08 (11.28%)	*	0.04 (2.46%)	0.02 (1.76%)	*	0.03 (3.06%)	0.02 (2.14%)	*
	NH_4^+	0.17 (28.31%)	0.10 (22.09%)	*	0.10 (27.57%)	0.07 (22.05%)	*	0.02 (4.03%)	0.01 (3.31%)	*	0.01 (3.75%)	0.01 (3.19%)	
	OM	0.03 (0.79%)	0.03 (1.07%)		0.02 (0.77%)	0.02 (0.73%)		0.01 (0.15%)	0.01 (0.20%)		0 (0.15%)	0 (0.14%)	
	BC	0 (0.12%)	0 (0.13%)		0 (0.18%)	0 (0.18%)		0 (0.01%)	0 (0.02%)		0 (0.03%)	0 (0.03%)	
**Transportation**	$PM_{2.5}$	0.50 (6.23%)	0.32 (4.91%)	*	0.34 (5.77%)	0.25 (4.53%)	*	0.10 (1.25%)	0.06 (0.98%)	*	0.07 (1.15%)	0.05 (0.91%)	*
	NO_3^-	0.10 (12.67%)	0.07 (11.10%)	*	0.08 (11.71%)	0.06 (10.16%)	*	0.02 (2.53%)	0.01 (2.22%)	*	0.02 (2.34%)	0.01 (2.03%)	*
	SO_4^{2-}	0.02 (1.29%)	0.01 (1.17%)	-	0.01 (1.06%)	0.01 (0.95%)		0 (0.26%)	0 (0.23%)		0 (0.21%)	0 (0.19%)	
	NH_4^+	0.03 (5.36%)	0.02 (4.06%)	*	0.02 (6.22%)	0.01 (4.81%)	*	0.01 (1.07%)	0 (0.81%)	*	0 (1.24%)	0 (0.96%)	
	OM	0.30 (7.31%)	0.25 (6.83%)	*	0.16 (5.85%)	0.14 (5.18%)	*	0.06 (1.46%)	0.05 (1.37%)	*	0.03 (1.17%)	0.03 (1.04%)	
	BC	0.10 (17.91%)	0.06 (14.66%)	*	0.06 (18.60%)	0.04 (15.18%)	*	0.02 (3.58%)	0.01 (2.93%)	*	0.01 (3.72%)	0.01 (3.04%)	
**RWC**	$PM_{2.5}$	0.52 (6.34%)	0.20 (2.96%)	*	0.53 (8.37%)	0.24 (4.38%)	*	0.10 (1.27%)	0.04 (0.59%)	*	0.11 (1.67%)	0.05 (0.88%)	*
	NO_3^-	0 (0.38%)	0 (0.20%)		0 (0.35%)	0 (0.15%)		0 (0.08%)	0 (0.04%)		0 (0.07%)	0 (0.03%)	
	SO_4^{2-}	n/a	n/a	-	0 (0.50%)	0 (0.60%)		n/a	n/a	-	0 (0.10%)	0 (0.12%)	
	NH_4^+	0 (0.18%)	0 (0.08%)		0 (0.40%)	0 (0.28%)		0 (0.04%)	0 (0.02%)		0 (0.08%)	0 (0.06%)	
	OM	0.62 (14.21%)	0.29 (7.36%)	*	0.55 (17.98%)	0.29 (10.17%)	*	0.12 (2.84%)	0.06 (1.47%)	*	0.11 (3.60%)	0.06 (2.03%)	*
	BC	0.03 (6.35%)	0.01 (3.80%)	*	0.04 (11.83%)	0.02 (7.45%)	*	0.01 (1.27%)	0 (0.76%)	*	0.01 (2.37%)	0 (1.49%)	*
**Mining**	$PM_{2.5}$	0.07 (0.90%)	0.07 (1.22%)		0.06 (1.03%)	0.06 (1.24%)		0.01 (0.18%)	0.01 (0.24%)		0.01 (0.21%)	0.01 (0.25%)	
	NO_3^-	0 (0.35%)	0 (0.99%)	*	0 (0.32%)	0 (0.89%)	*	0 (0.07%)	0 (0.20%)		0 (0.06%)	0 (0.18%)	
	SO_4^{2-}	0.06 (3.75%)	0.06 (5.09%)		0.05 (5.94%)	0.05 (6.11%)		0.01 (0.75%)	0.01 (1.02%)		0.01 (1.19%)	0.01 (1.22%)	
	NH_4^+	0.01 (1.86%)	0.01 (2.24%)	*	0.01 (2.60%)	0.01 (2.64%)	*	0 (0.37%)	0 (0.45%)	*	0 (0.52%)	0 (0.53%)	
	OM	0 (0.01%)	0 (0.03%)		0 (0.02%)	0 (0.05%)		n/a	n/a	-	n/a	0 (0.01%)	*
	BC	0 (0.01%)	0 (0.04%)		0 (0.03%)	0 (0.07%)		n/a	n/a	-	0 (0.01%)	0 (0.01%)	

Note: BC- Black carbon; NH_4^+- Ammonium; NO_3^-- Nitrate; OM- Organic matter; RWC – Residential wood combustion; SO_4^{2-}—Sulfate. “Sig” is significance based on $p < 0.05$.

Table 11S. Estimated reduction in exposure of total mass $PM_{2.5}$ and its five major components for males versus females groups by four selected emission sectors and combined for 100% and 20% scenarios between 2007 and 2016.

		100% reduction ($\mu g/m^3$)						20% reduction ($\mu g/m^3$)					
		Male	Female	Sig.	Male	Female	Sig.	Male	Female	Sig.	Male	Female	Sig.
**Sector**	**Component**	**2007**			**2016**			**2007**			**2016**		
**All 4 sectors combined**	$PM_{2.5}$	2.10 (26.05%)	2.10 (26.07%)		1.64 (27.07%)	1.65 (27.08%)		0.36 (4.45%)	0.36 (4.45%)		0.28 (4.56%)	0.28 (4.57%)	
	NO_3^-	0.42 (53.16%)	0.42 (53.12%)		0.32 (45.12%)	0.32 (45.05%)		0.06 (7.60%)	0.06 (7.60%)		0.04 (6.21%)	0.04 (6.21%)	
	SO_4^{2-}	0.20 (13.77%)	0.20 (13.73%)		0.18 (20.55%)	0.18 (20.47%)		0.04 (2.94%)	0.04 (2.93%)		0.03 (3.81%)	0.03 (3.80%)	
	NH_4^+	0.22 (35.46%)	0.22 (35.42%)		0.13 (36.49%)	0.13 (36.43%)		0.03 (5.42%)	0.03 (5.41%)		0.02 (5.40%)	0.02 (5.40%)	
	OM	0.93 (22.02%)	0.94 (22.09%)		0.72 (24.19%)	0.72 (24.27%)		0.19 (4.41%)	0.19 (4.43%)		0.14 (4.84%)	0.14 (4.86%)	
	BC	0.13 (24.16%)	0.13 (24.17%)		0.10 (30.27%)	0.10 (30.28%)		0.03 (4.83%)	0.03 (4.84%)		0.02 (6.06%)	0.02 (6.06%)	
**Agriculture**	$PM_{2.5}$	1.03 (12.78%)	1.03 (12.75%)		0.73 (12.17%)	0.73 (12.12%)		0.15 (1.83%)	0.15 (1.82%)		0.10 (1.64%)	0.10 (1.64%)	
	NO_3^-	0.33 (40.55%)	0.33 (40.49%)		0.24 (33.50%)	0.24 (33.41%)		0.04 (5.22%)	0.04 (5.21%)		0.03 (3.93%)	0.03 (3.92%)	
	SO_4^{2-}	0.16 (11.26%)	0.16 (11.21%)		0.13 (15.16%)	0.13 (15.06%)		0.03 (2.44%)	0.03 (2.43%)		0.03 (3.02%)	0.03 (3.01%)	
	NH_4^+	0.17 (28.10%)	0.17 (28.04%)		0.09 (27.35%)	0.09 (27.27%)		0.02 (4.00%)	0.02 (4.00%)		0.01 (3.73%)	0.01 (3.72%)	
	OM	0.03 (0.80%)	0.03 (0.79%)		0.02 (0.77%)	0.02 (0.76%)		0.01 (0.15%)	0.01 (0.15%)		0 (0.15%)	0 (0.15%)	
	BC	0 (0.12%)	0 (0.12%)		0 (0.19%)	0 (0.18%)		0 (0.01%)	0 (0.01%)		0 (0.03%)	0 (0.03%)	
**Transportation**	$PM_{2.5}$	0.49 (6.17%)	0.50 (6.18%)		0.34 (5.70%)	0.34 (5.71%)		0.10 (1.23%)	0.10 (1.24%)		0.07 (1.14%)	0.07 (1.14%)	
	NO_3^-	0.09 (12.61%)	0.09 (12.63%)		0.08 (11.64%)	0.08 (11.64%)		0.02 (2.52%)	0.02 (2.53%)		0.02 (2.33%)	0.02 (2.33%)	
	SO_4^{2-}	0.02 (1.29%)	0.02 (1.29%)		0.01 (1.06%)	0.01 (1.05%)		0 (0.26%)	0 (0.26%)		0 (0.21%)	0 (0.21%)	
	NH_4^+	0.03 (5.31%)	0.03 (5.32%)		0.02 (6.15%)	0.02 (6.16%)		0.01 (1.06%)	0.01 (1.06%)		0 (1.23%)	0 (1.23%)	
	OM	0.30 (7.29%)	0.30 (7.28%)		0.16 (5.81%)	0.16 (5.81%)		0.06 (1.46%)	0.06 (1.46%)		0.03 (1.16%)	0.03 (1.16%)	
	BC	0.10 (17.78%)	0.10 (17.77%)		0.06 (18.43%)	0.06 (18.40%)		0.02 (3.56%)	0.02 (3.55%)		0.01 (3.69%)	0.01 (3.68%)	
**RWC**	$PM_{2.5}$	0.50 (6.19%)	0.51 (6.23%)		0.51 (8.17%)	0.51 (8.21%)		0.10 (1.24%)	0.10 (1.25%)		0.10 (1.63%)	0.10 (1.64%)	
	NO_3^-	0 (0.37%)	0 (0.37%)		0 (0.34%)	0 (0.34%)		0 (0.07%)	0 (0.07%)		0 (0.07%)	0 (0.07%)	
	SO_4^{2-}	n/a	n/a		0 (0.51%)	0 (0.51%)		n/a	n/a		0 (0.10%)	0 (0.10%)	
	NH_4^+	0 (0.18%)	0 (0.18%)		0 (0.39%)	0 (0.39%)		0 (0.04%)	0 (0.04%)		0 (0.08%)	0 (0.08%)	
	OM	0.61 (13.92%)	0.61 (14.01%)		0.54 (17.59%)	0.54 (17.67%)		0.12 (2.78%)	0.12 (2.80%)		0.11 (3.52%)	0.11 (3.53%)	
	BC	0.03 (6.25%)	0.03 (6.27%)		0.04 (11.63%)	0.04 (11.66%)		0.01 (1.25%)	0.01 (1.25%)		0.01 (2.33%)	0.01 (2.33%)	
**Mining**	$PM_{2.5}$	0.07 (0.91%)	0.07 (0.91%)		0.06 (1.04%)	0.06 (1.04%)		0.01 (0.18%)	0.01 (0.18%)		0.01 (0.21%)	0.01 (0.21%)	
	NO_3^-	0 (0.38%)	0 (0.38%)		0 (0.35%)	0 (0.35%)		0 (0.08%)	0 (0.08%)		0 (0.07%)	0 (0.07%)	
	SO_4^{2-}	0.06 (3.80%)	0.06 (3.81%)		0.05 (5.94%)	0.05 (5.96%)		0.01 (0.76%)	0.01 (0.76%)		0.01 (1.19%)	0.01 (1.19%)	
	NH_4^+	0.01 (1.87%)	0.01 (1.87%)		0.01 (2.60%)	0.01 (2.61%)		0 (0.37%)	0 (0.37%)		0 (0.52%)	0 (0.52%)	
	OM	0 (0.01%)	0 (0.01%)		0 (0.03%)	0 (0.03%)		n/a	n/a	-	0 (0.01%)	0 (0.01%)	
	BC	0 (0.01%	0 (0.01%)		0 (0.04%)	0 (0.03%)		n/a	n/a	-	0 (0.01%)	0 (0.01%)	

Note: BC- Black carbon; NH_4^+- Ammonium; NO_3^-- Nitrate; OM- Organic matter; RWC – Residential wood combustion; SO_4^{2-}—Sulfate. "Sig" is significance based on $p < 0.05$.

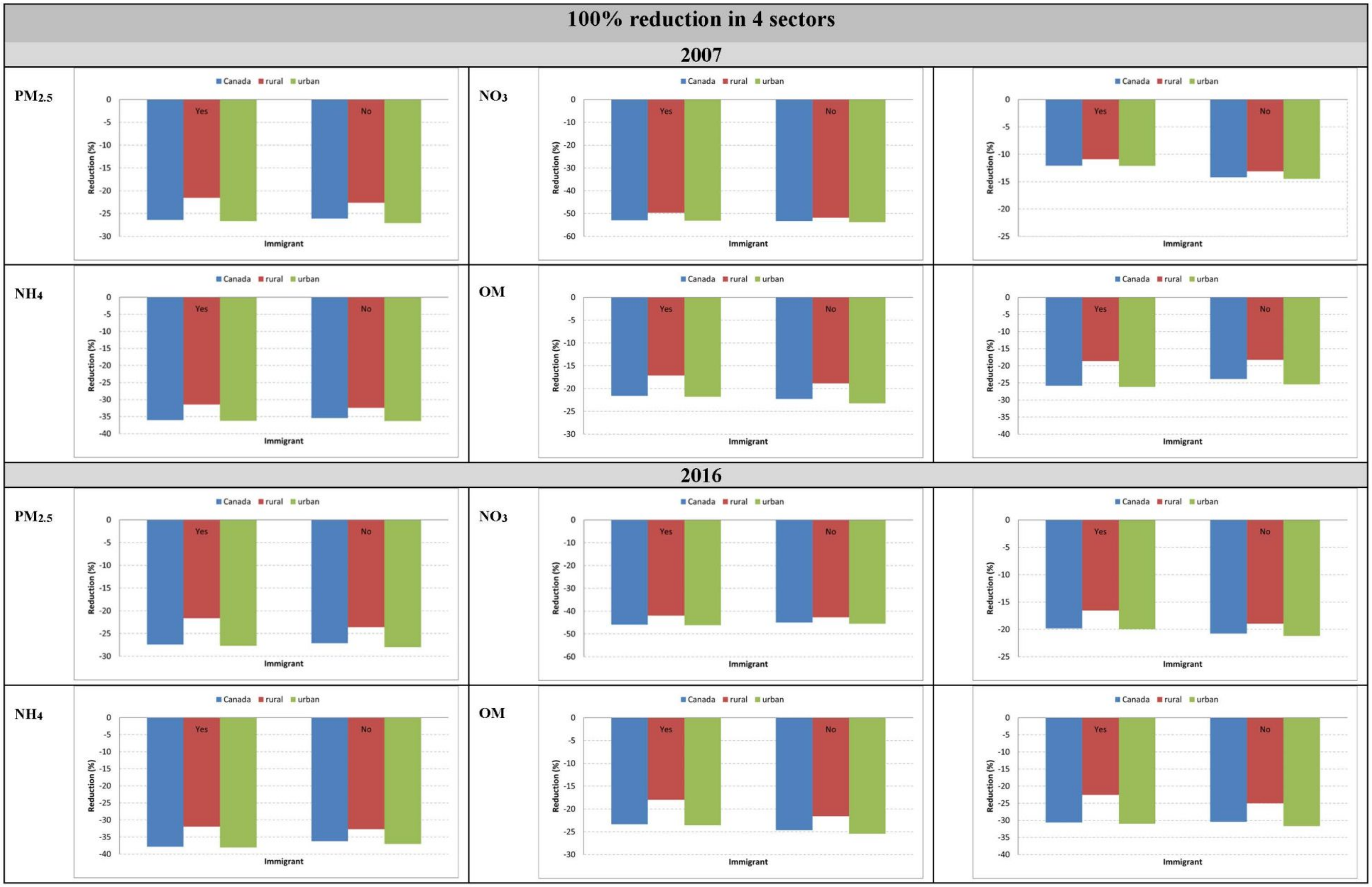

Figure 2S. Estimated percent reduction in $PM_{2.5}$ components following complete emission removal from four sectors in Canada (2007 versus 2016): Differences across Immigrant status and Urban-Rural populations.

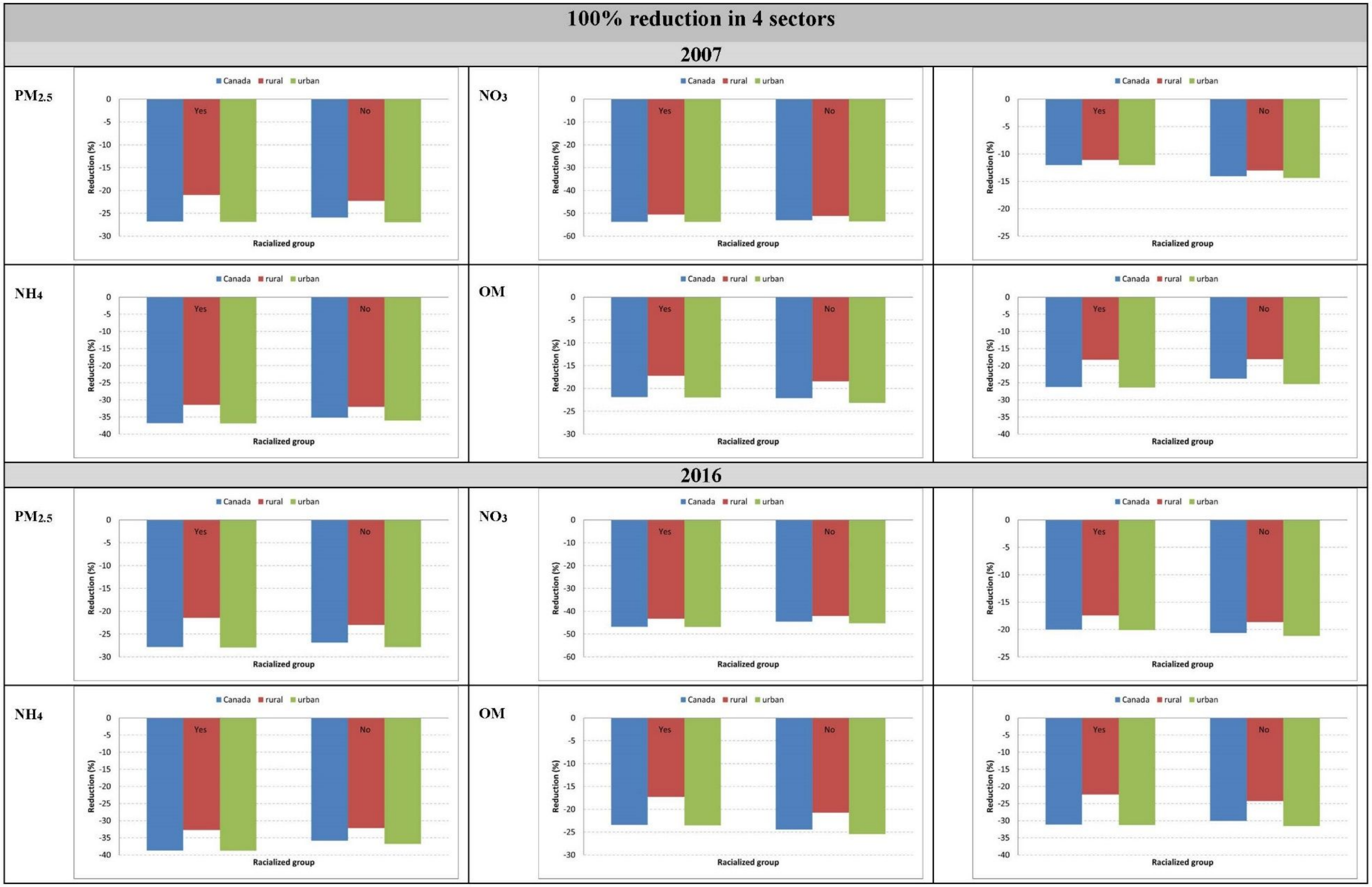

Figure 3S. Estimated percent reduction in $PM_{2.5}$ components following complete emission removal from four sectors in Canada (2007 versus 2016): Differences across people categorized by Race and Urban-Rural populations.

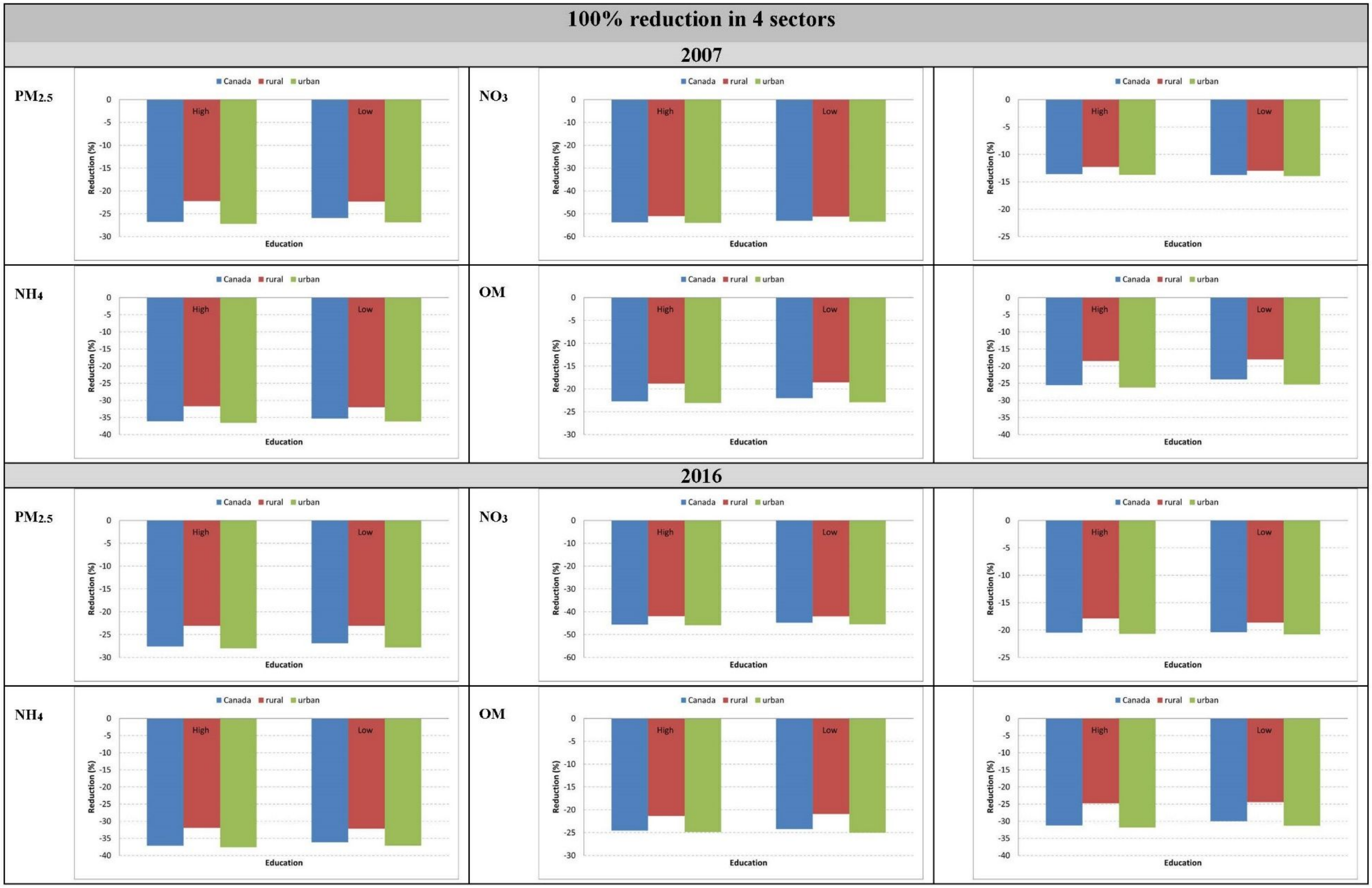

Figure 4S: Estimated percent reduction in $PM_{2.5}$ components following complete emission removal from four sectors in Canada (2007 versus 2016): Differences across two Educational Levels and Urban-Rural populations.

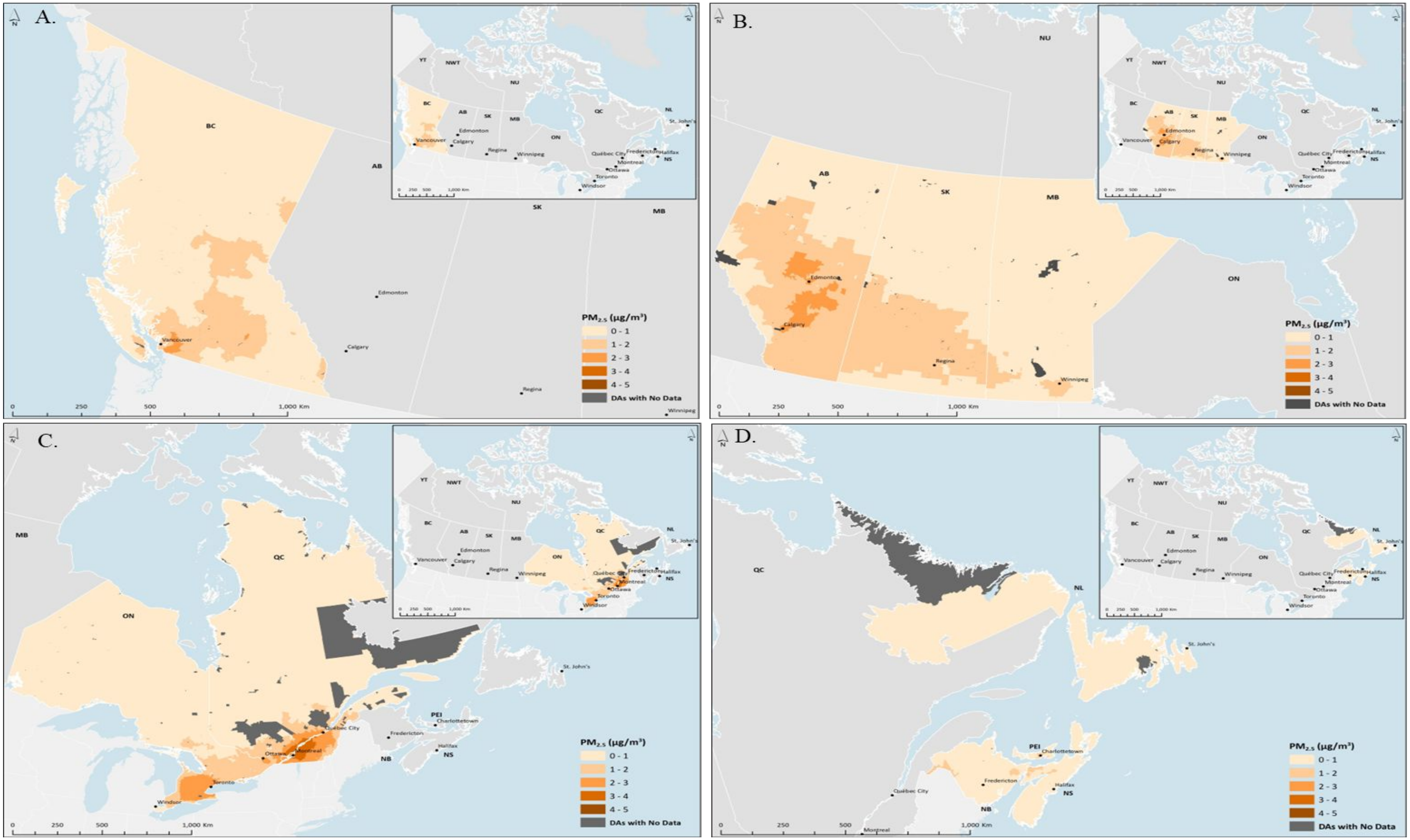

Figure 5S. 100% policy reduction scenario of fine particulate matter ($PM_{2.5}$) in four major emission sectors combined (i.e., agriculture, transportation, residential wood combustion, and mining) captured in Western (A), Prairies (B), Central (C), and Atlantic (D) regions during 2007.

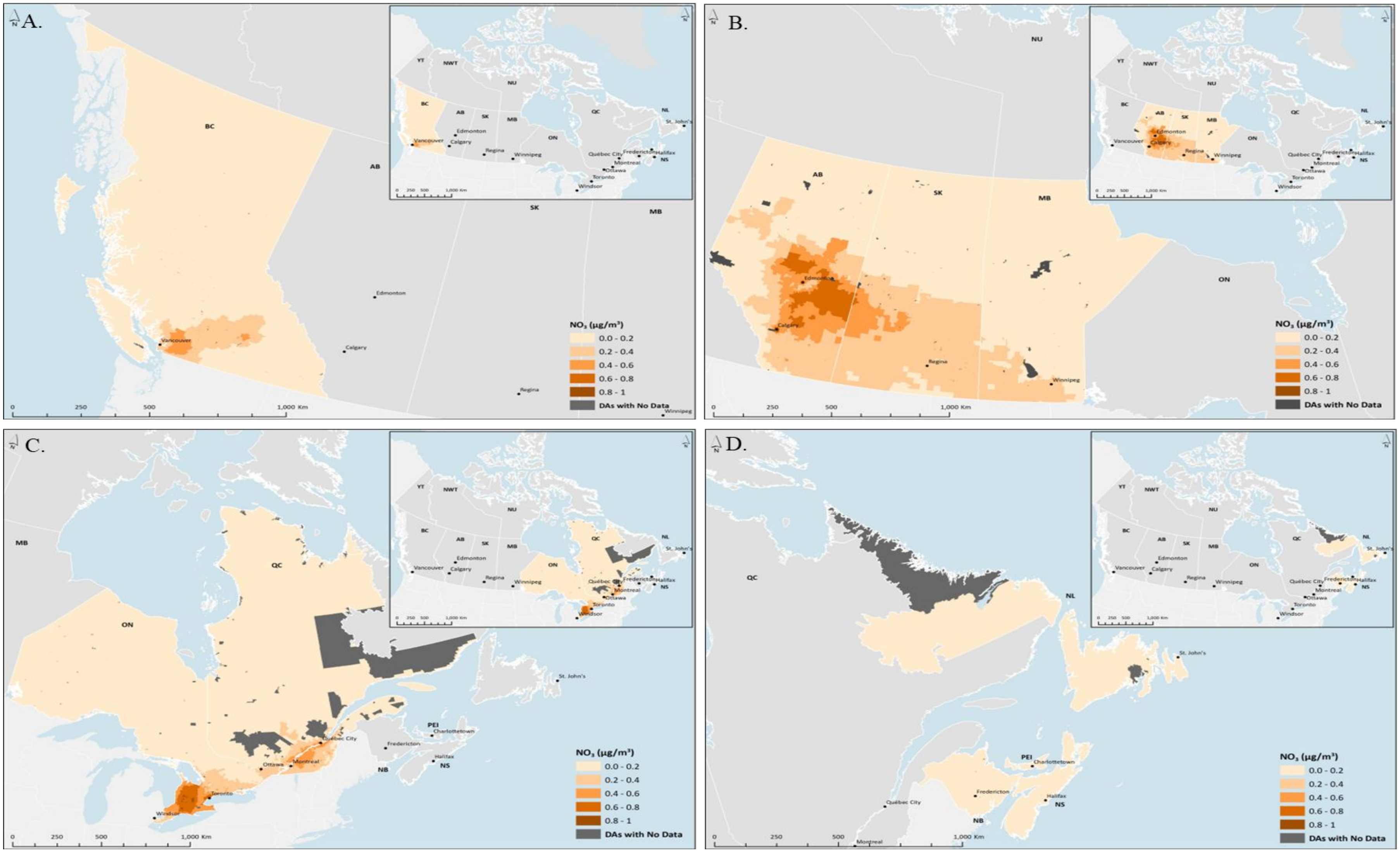

Figure 6S. 100% policy reduction scenario of nitrate (NO_3^-) in four major emission sectors combined (i.e., agriculture, transportation, residential wood combustion, and mining) captured in Western (A), Prairies (B), Central (C), and Atlantic (D) regions during 2007.

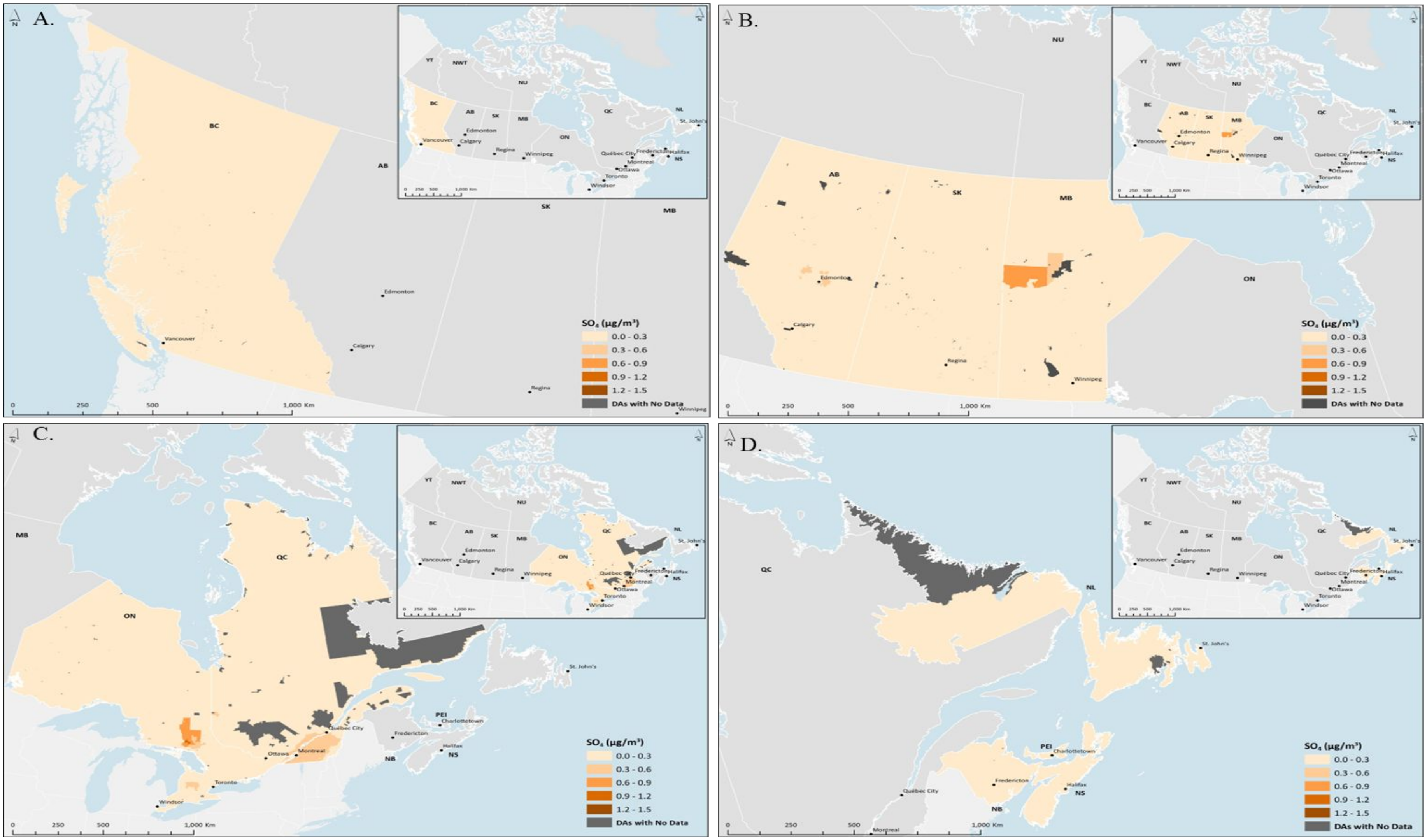

Figure 7S. 100% policy reduction scenario of sulfate $(SO_4{}^{2})^-$ in four major emission sectors combined (i.e., agriculture, transportation, residential wood combustion, and mining) captured in Western (A), Prairies (B), Central (C), and Atlantic (D) regions during 2007.

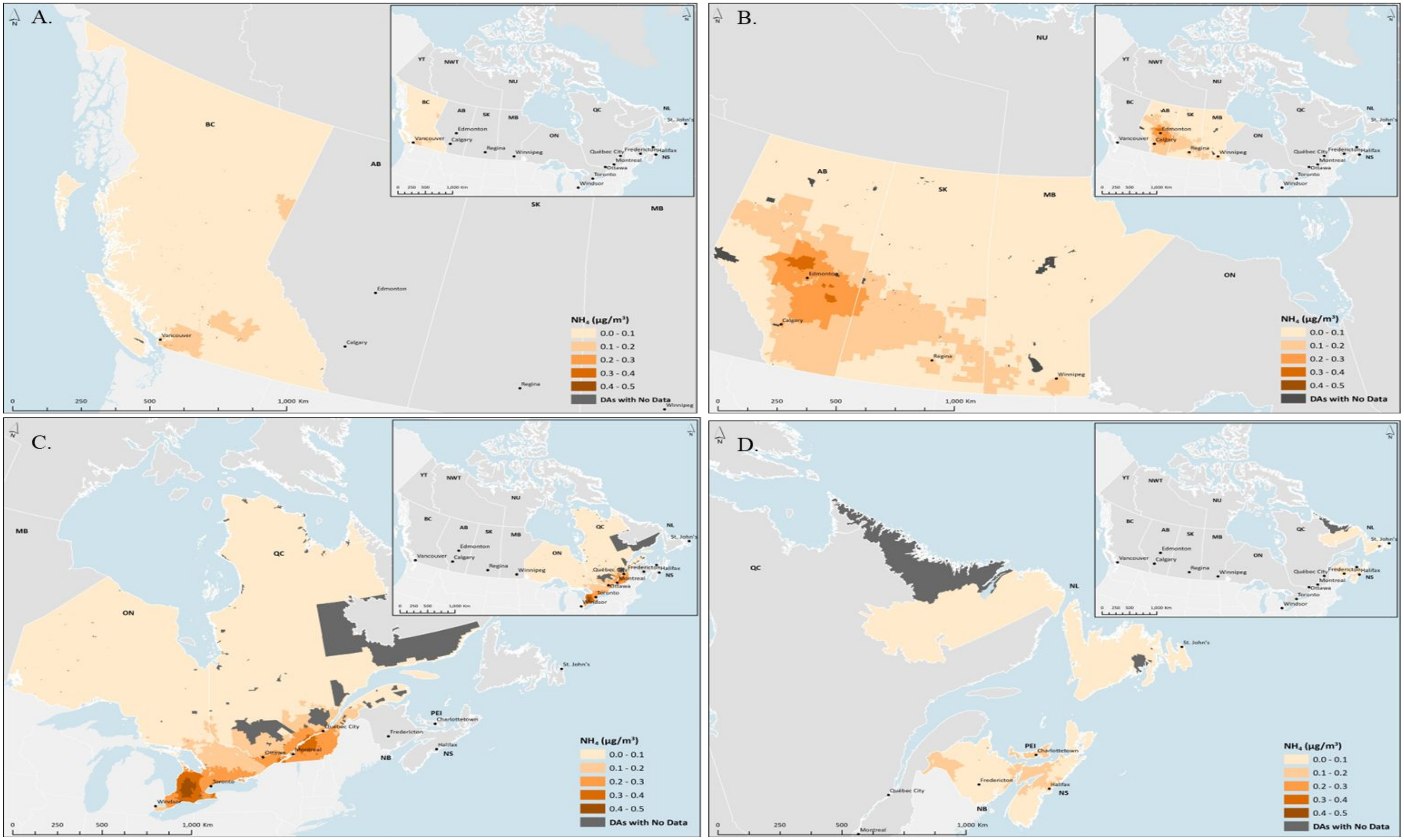

Figure 8S. 100% policy reduction scenario of ammonium (NH_4^+) in four major emission sectors combined (i.e., agriculture, transportation, residential wood combustion, and mining) captured in Western (A), Prairies (B), Central (C), and Atlantic (D) regions during 2007.

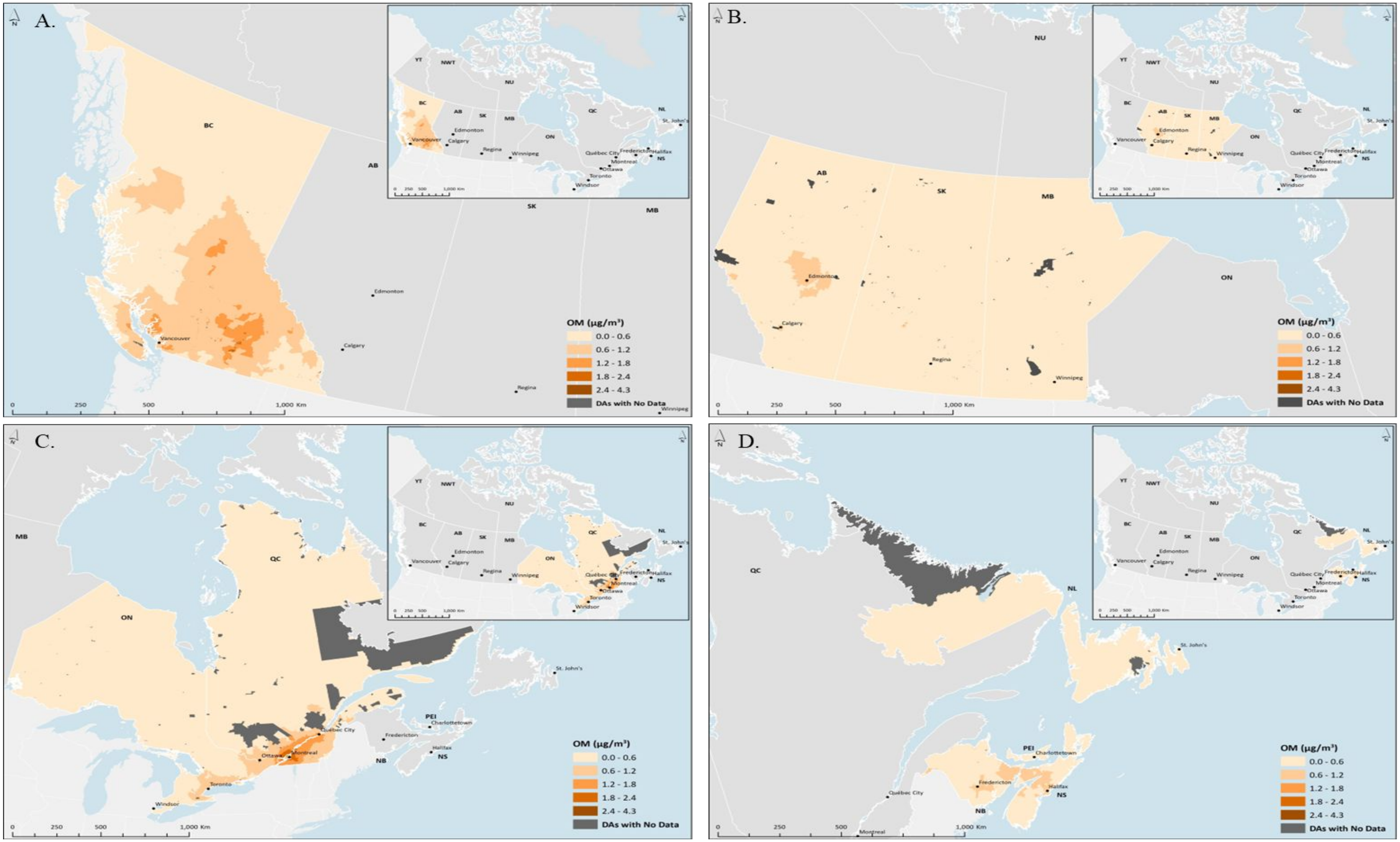

Figure 9S. 100% policy reduction scenario of organic matter (OM) in four major emission sectors combined (i.e., agriculture, transportation, residential wood combustion, and mining) captured in Western (A), Prairies (B), Central (C), and Atlantic (D) regions during 2007.

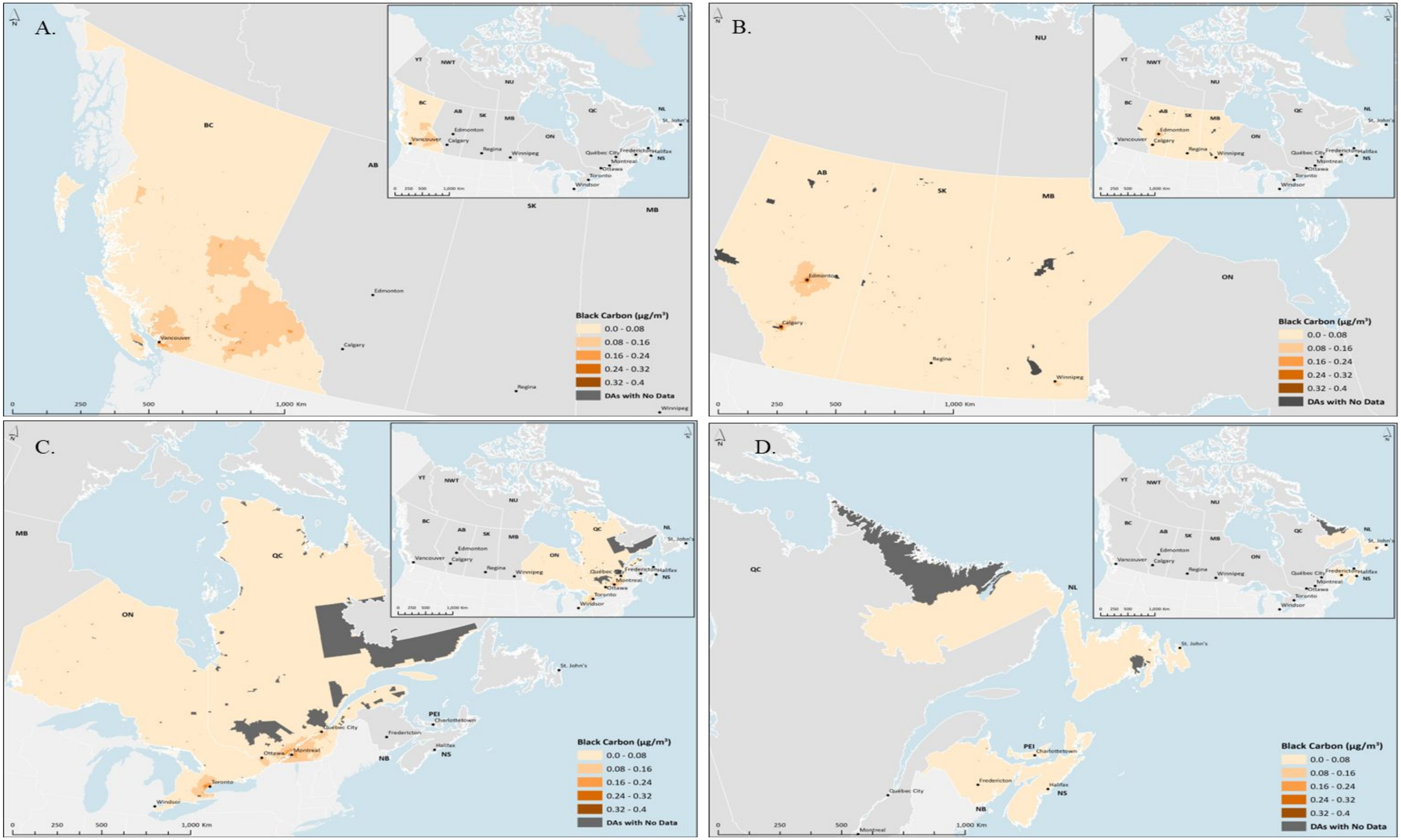

Figure 10S. 100% policy reduction scenario of black carbon (BC) in four major emission sectors combined (i.e., agriculture, transportation, residential wood combustion, and mining) captured in Western (A), Prairies (B), Central (C), and Atlantic (D) regions during 2007.

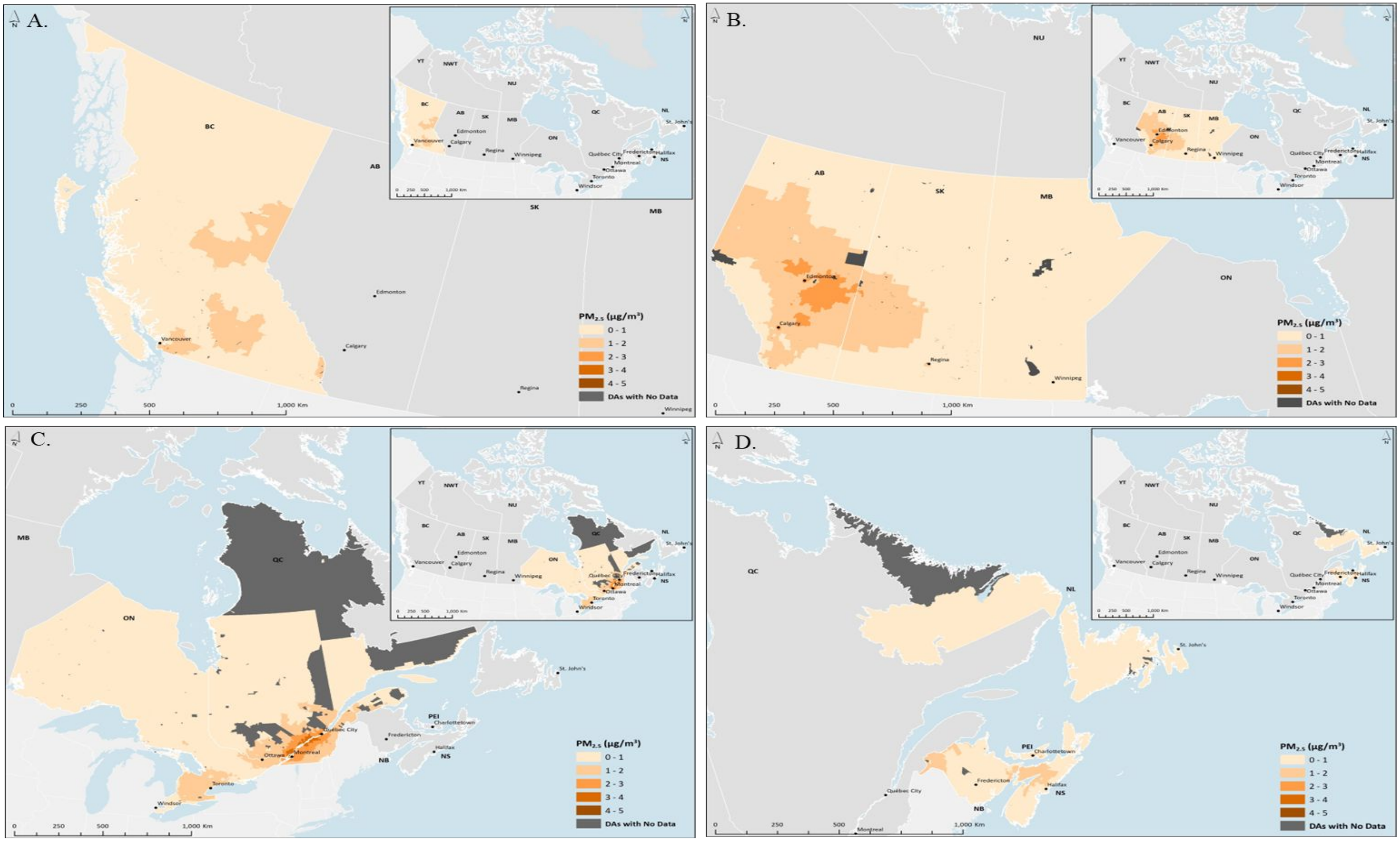

Figure 11S. 100% policy reduction scenario of fine particulate matter ($PM_{2.5}$) in four major emission sectors combined (i.e., agriculture, transportation, residential wood combustion, and mining) captured in Western (A), Prairies (B), Central (C), and Atlantic (D) regions during 2016.

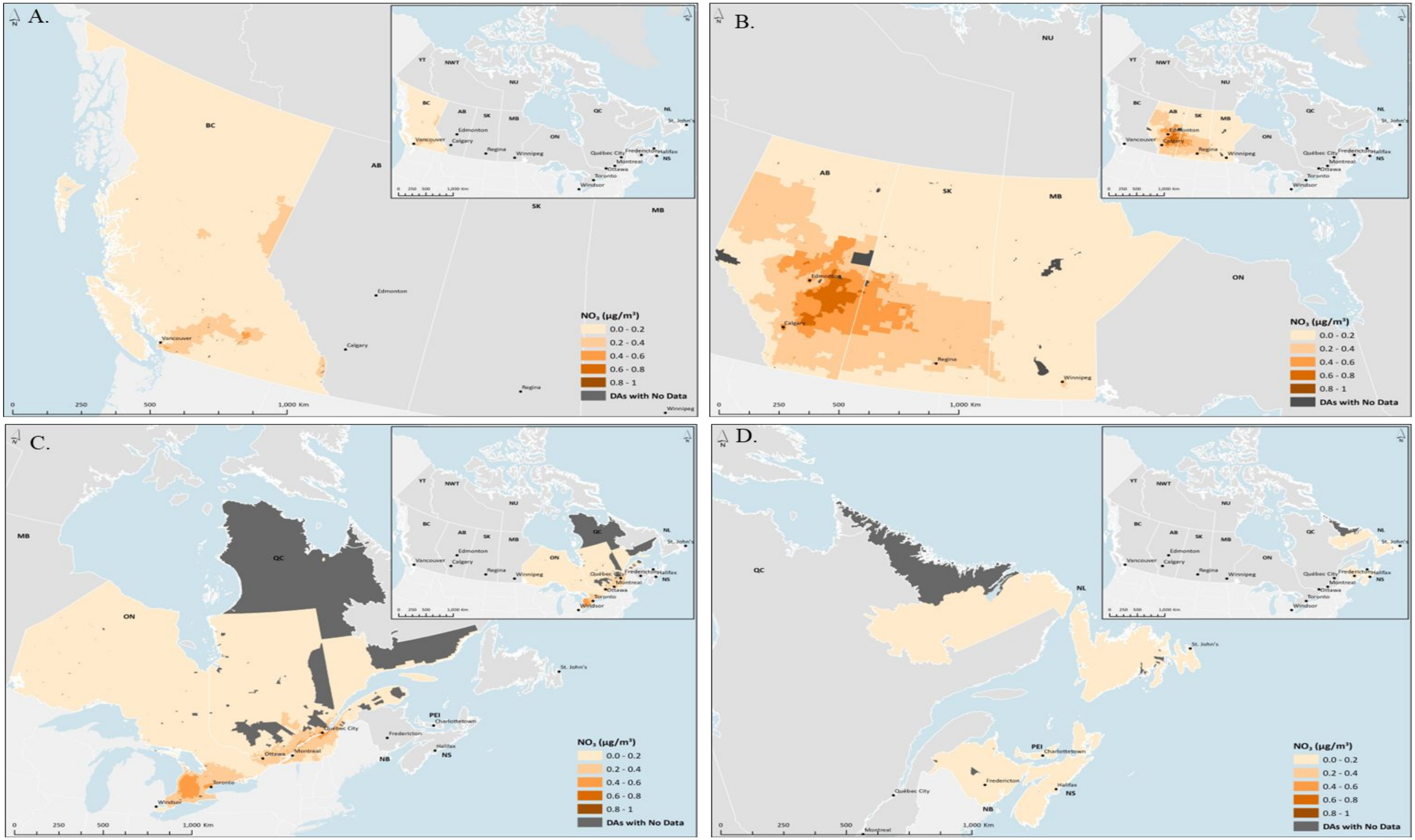

Figure 12S. 100% policy reduction scenario of nitrate (NO_3^-) in four major emission sectors combined (i.e., agriculture, transportation, residential wood combustion, and mining) captured in Western (A), Prairies (B), Central (C), and Atlantic (D) regions during 2016.

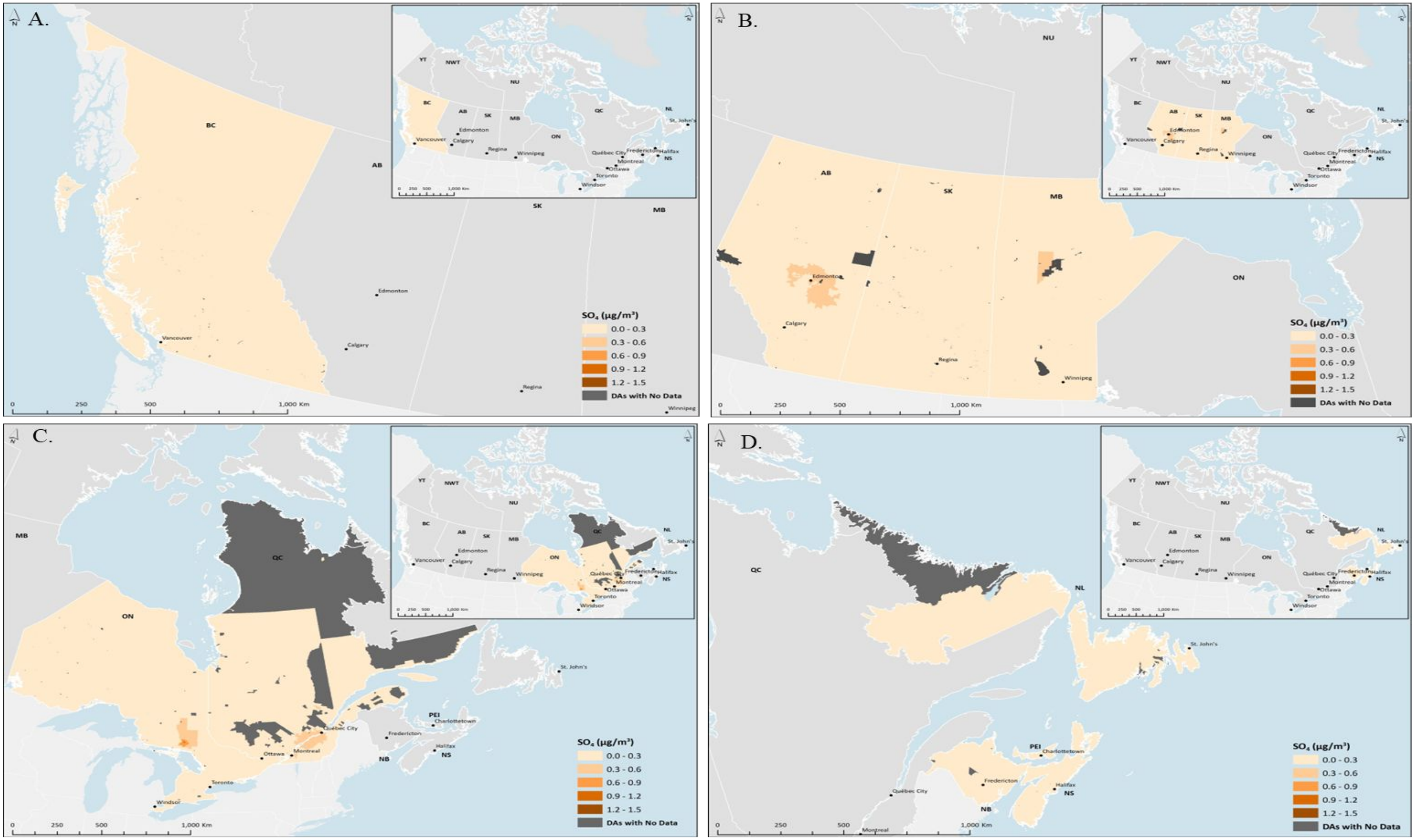

Figure 13S. 100% policy reduction scenario of sulfate (SO_4^{2-}) in four major emission sectors combined (i.e., agriculture, transportation, residential wood combustion, and mining) captured in Western (A), Prairies (B), Central (C), and Atlantic (D) regions during 2016.

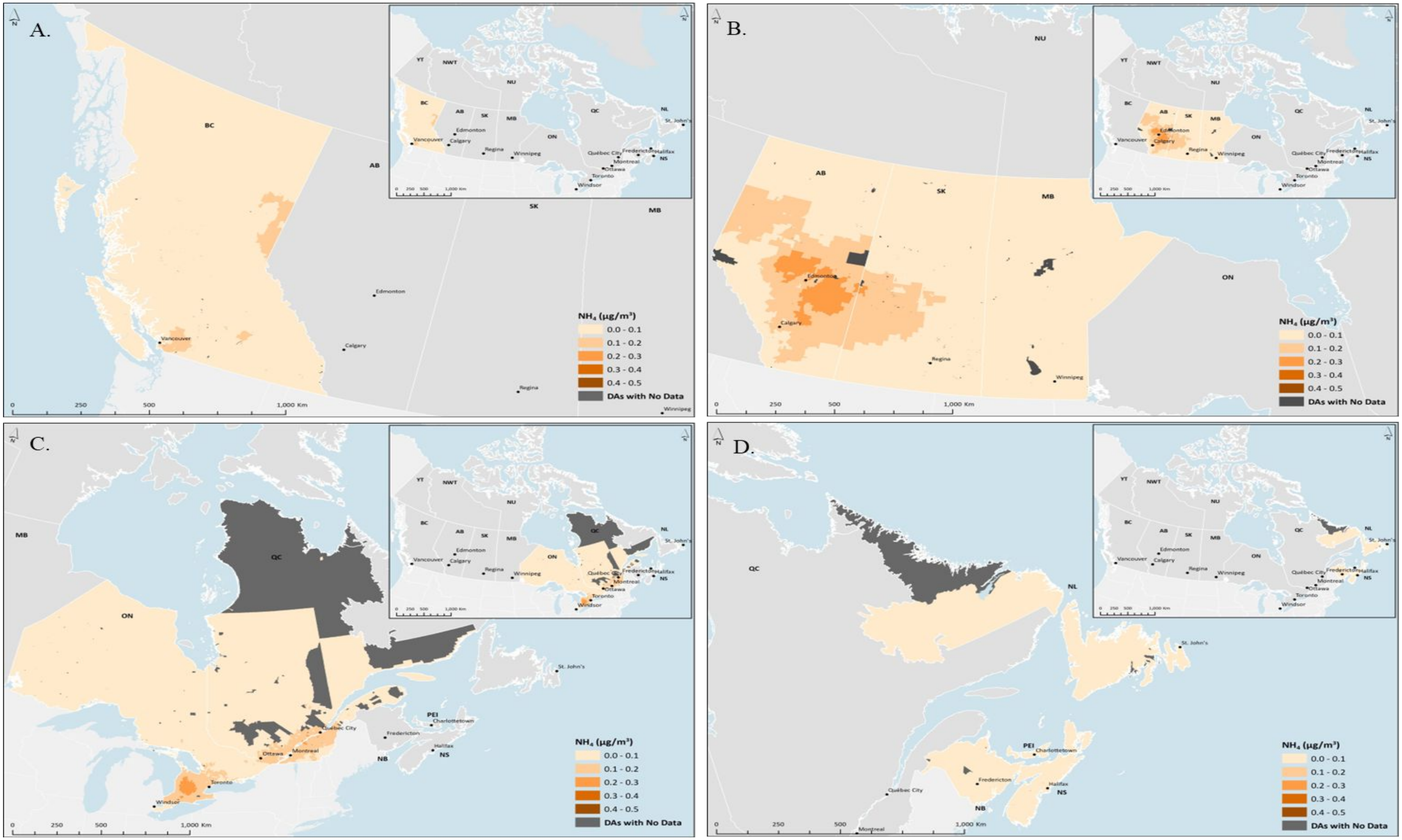

Figure 14S. 100% policy reduction scenario of ammonium (NH_4^+) in four major emission sectors combined (i.e., agriculture, transportation, residential wood combustion, and mining) captured Western (A), Prairies (B), Central Quebec (C), and Atlantic (D) regions during 2016.

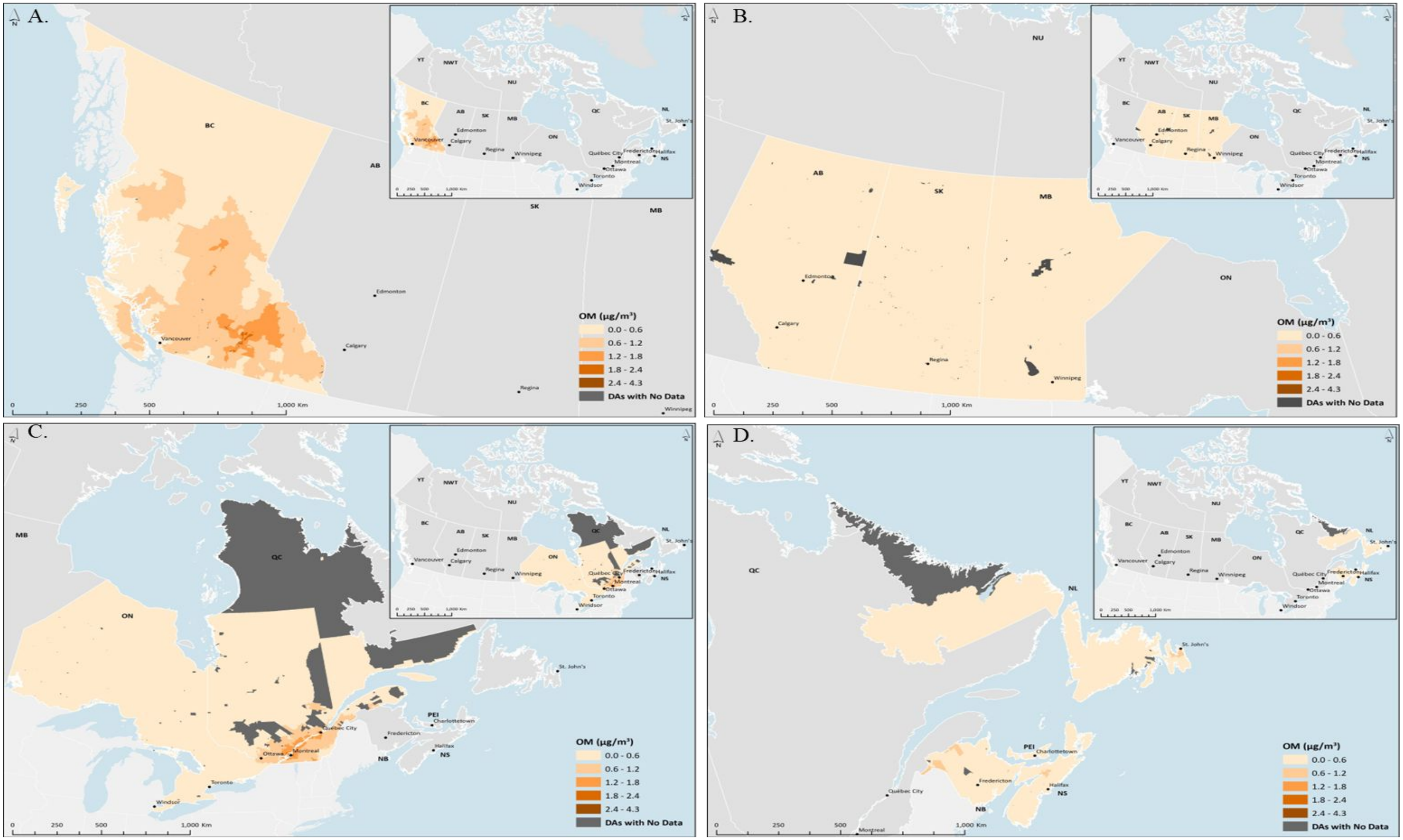

Figure 15S. 100% policy reduction scenario of organic matter (OM) in four major emission sectors combined (i.e., agriculture, transportation, residential wood combustion, and mining) captured in Western (A), Prairies (B), Central (C), and Atlantic (D) regions during 2016.

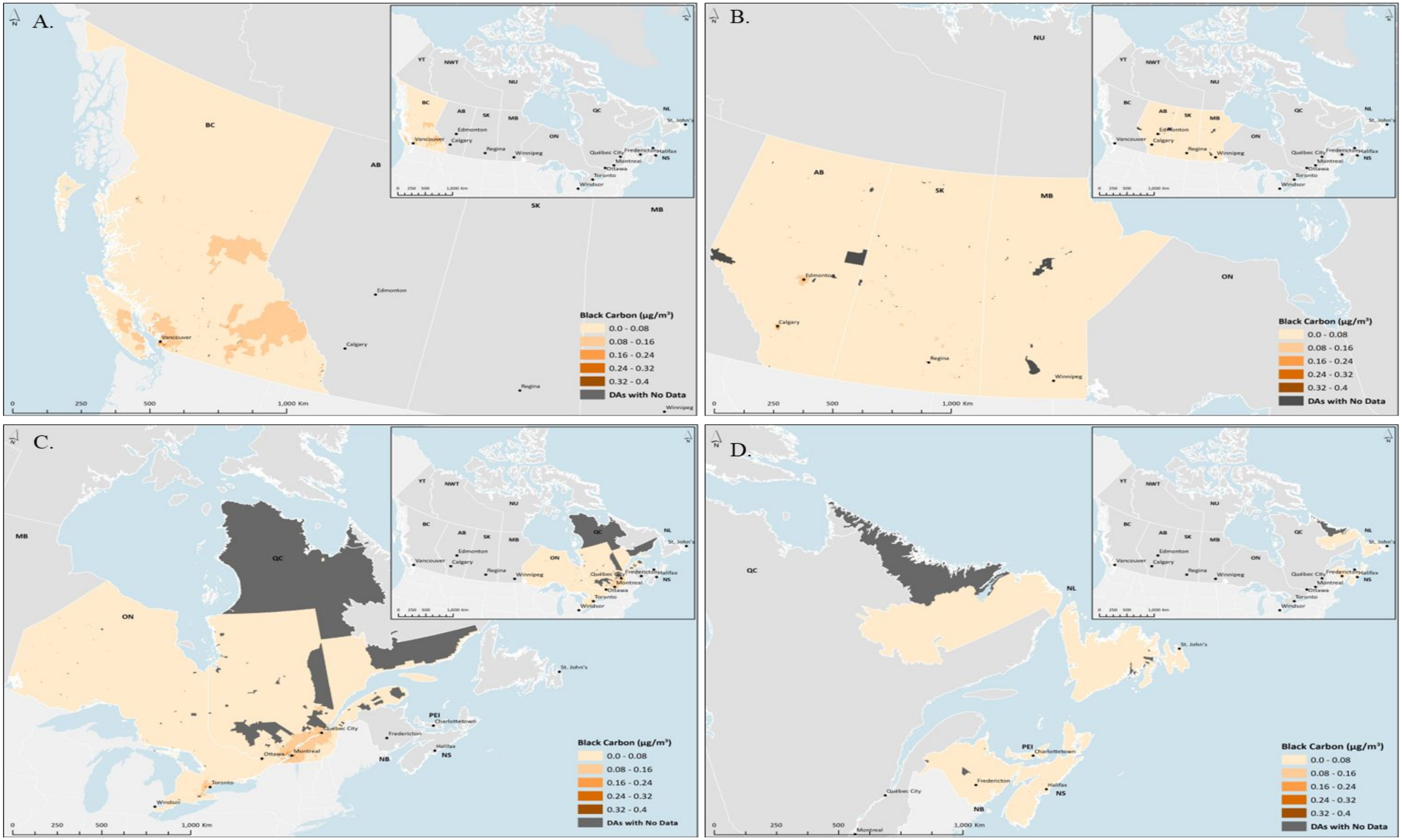

Figure 16S. 100% policy reduction scenario of black carbon (BC) in four major emission sectors combined (i.e., agriculture, transportation, residential wood combustion, and mining) captured in Western (A), Prairies (B), Central (C), and Atlantic (D) regions during 2016.
